# Supra-biomimetic Impact-Resistant Composites via Harnessing Macro–Microscale Competition

**DOI:** 10.34133/research.1358

**Published:** 2026-07-10

**Authors:** Miao Lei, Mengqi Sun, Qixuan Zhu, Zihan Hao, Dehua Tan, Chaohui Wu, Yueying Yang, Chaohong Zhang, Xuewen Wang, Wei Huang, Qianbo Lu

**Affiliations:** ^1^ State Key Laboratory of Flexible Electronics (LoFE) and Institute of Flexible Electronics (IFE), MIIT Key Laboratory of Flexible Electronics (KLoFE), Shaanxi Key Laboratory of Flexible Electronics, Northwestern Polytechnical University, Xi’an, China.; ^2^ Key Laboratory of Flexible Electronics of Zhejiang Province, Ningbo Institute of Northwestern Polytechnical University, Ningbo, China.; ^3^ School of Automation, Northwestern Polytechnical University, Xi’an, China.; ^4^ School of Flexible Electronics and State Key Laboratory of Optoelectronic Materials and Technologies, Sun Yat-sen University, Shenzhen, China.

## Abstract

Natural biomaterials achieve exceptional mechanical performance through multi-level hierarchical architectures, yet replicating or surpassing such topological control and multiscale synergy in synthetic hydrogels remains challenging. A key obstacle is the often-overlooked macro–microscale competition mechanism, where macroscopic reinforcement can conflict with finer-scale energy-dissipation pathways. Here, we overcome this limitation by embedding a 3-dimensionally printed gradient-twisted plywood (GT) framework into a hierarchically anisotropic (HA) hydrogel matrix, creating a topologically controllable supra-biomimetic composite (GT-HA composite). A supra-biomimetic design strategy is employed to regulate the macro–microscale competition, wherein the GT framework is expressly designed to coordinate macroscale stress guidance and crack deflection with micrometer-, nanometer-, and molecular-scale dissipation pathways. This coordinated multiscale dissipation endows composites with superior impact resistance. The GT-HA composite attenuates up to 88% of impact force at a low velocity and achieves a compressive strength of 183.57 MPa at a large strain rate of around 4,000 s^−1^ while maintaining long-term stability (<5% decay over 35 d). Notably, the fabrication process is compatible with integrated circuit/microelectromechanical system technologies, allowing wafer-level integration that effectively protects high-value devices such as processor dies and flexible circuits under high-speed impact. This work establishes a scalable strategy for designing ultra-impact-resistant materials by actively harnessing macro–micro competition, with promising applications in embodied intelligence, aerospace, and advanced electronics protection.

## Introduction

Protection against mechanical impact represents a pervasive challenge spanning diverse fields, including sports safety [1,2], electronic packaging [3], and aerospace applications [4,5]. Conventional impact-protective materials, such as metals, foams, and polymers, primarily rely on their intrinsic energy-absorbing buffering effects or strain resistance to mitigate impacts [6,7]. However, increasingly stringent safety standards call for materials with more comprehensive and efficient impact-protection performance [8,9]. Certain natural materials simultaneously exhibit high strength, toughness, and impact resistance, which stem primarily from their intricate hierarchical structures that operate as multiscale energy-dissipation systems [10–12]. A paradigmatic example is the dactyl club of *Odontodactylus scyllarus*, whose periodic regions feature a spatially hierarchical twisted plywood structure composed of spirally arranged chitin fiber bundles. Upon impact, this architecture triggers crack deflection for dynamic toughening, effectively dispersing stress waves and exhibiting an exceptional energy-absorption capacity [13–15]. Another paradigm is bamboo, whose gradient structure enables stepwise energy dissipation via gradient stress transfer [16–18]: the dense and rigid outer fiber layer resists impact stress concentration via high stiffness, while the flexible and porous inner parenchyma cells dissipate energy via plastic deformation. These biological blueprints offer a compelling strategy for designing high-performance protective materials, particularly for soft matter systems that emulate natural structural organization. Hydrogels, in this context, serve as an ideal platform for implementing such biomimetic design [19–21]. For instance, inspired by *O. scyllarus*, Tang et al. [22] developed a high-toughness and impact-resistant hydrogel by synergizing a long-range-ordered cellulose nanocrystal assembly, distinct nanocrystalline domains, and dynamic interfacial interactions. Similarly, Xu et al. [23] introduced multiscale orientation and gradient crystalline cross-linked networks into polyvinyl alcohol (PVA)/cellulose nanofiber composite gels, constructing a multi-level oriented heterogeneous core–sheath hydrogel that resolves the conventional strength–toughness trade-off in hydrogel systems.

Despite substantial progress in the development of biomimetic hydrogels, the hierarchical complexity and spatial precision of their assembled structures remain inferior to those of natural biological tissues, largely due to the absence of deliberately designable, biomimetic topological frameworks. This limitation critically restricts their deployment in applications demanding high impact resistance, superior toughness, and long-term stability. Conventional fabrication approaches, such as mold casting and self-assembly techniques [24,25], typically yield simple geometries or ordered structures at the micrometer and nanometer scale, making it difficult to realize deliberate topological frameworks with a high degree of spatial freedom and tunable characteristics. While micro-/nanofabrication techniques (e.g., integrated circuit [IC]/microelectromechanical system [MEMS] processes) [26] enable accurate topological patterning at small scales, they are impractical for constructing macroscopic architectures. Moreover, integrating such frameworks with hydrogel matrices usually relies on heterogeneous interfacial bonding, which introduces risks of delamination. Therefore, a manufacturing route that achieves multiscale, high-precision fabrication and ensures robust interfacial integration with hydrogels is essential for realizing deliberate biomimetic topological constructs.

Beyond topological framework design, the mechanical performance of biomimetic hydrogels can be further enhanced through the synergistic activation of multiscale energy-dissipation mechanisms. Strategies categorized as molecular engineering and structural engineering [27] have been demonstrated to boost energy dissipation effectively. Molecular engineering, including the construction of dual/multi-networks [28–30], the introduction of noncovalent interactions [31,32], and the formation of nanocrystalline domains [33,34], has proven effective in improving fracture toughness, strength, self-healing, and modulus. Parallel approaches in structural engineering, such as freeze-casting [35,36], mechanical training [37], and the Hofmeister effect [38], enable the creation of anisotropic micro-/nanostructures. When combined, molecular and structural engineering can orchestrate ordered hierarchical architectures across molecular, microscopic, and mesoscopic scales, leading to the cross-scale enhancement of mechanical properties [39–41]. However, existing research typically spans only 2 or 3 hierarchical levels, which limits further gains in energy-dissipation efficiency. More fundamentally, the introduction of a macroscopic topological framework does not guarantee enhanced performance. Instead, we identify a macro–microscale competition (MMC) mechanism, wherein the topological reinforcement at the macroscale can compete with, rather than synergize with, the energy-dissipation mechanisms at the microscale. This competition explains why simply adding a framework may even degrade overall mechanical properties, and it underscores the critical need to deliberately design the framework’s architecture to orchestrate the multiscale dissipation pathways. Therefore, the development of a hydrogel system that seamlessly integrates programmable topological frameworks with multiscale processing to activate synergistic energy dissipation, thereby surpassing the impact resistance of existing biomimetic hydrogels, remains challenging.

**Fig. 1.**
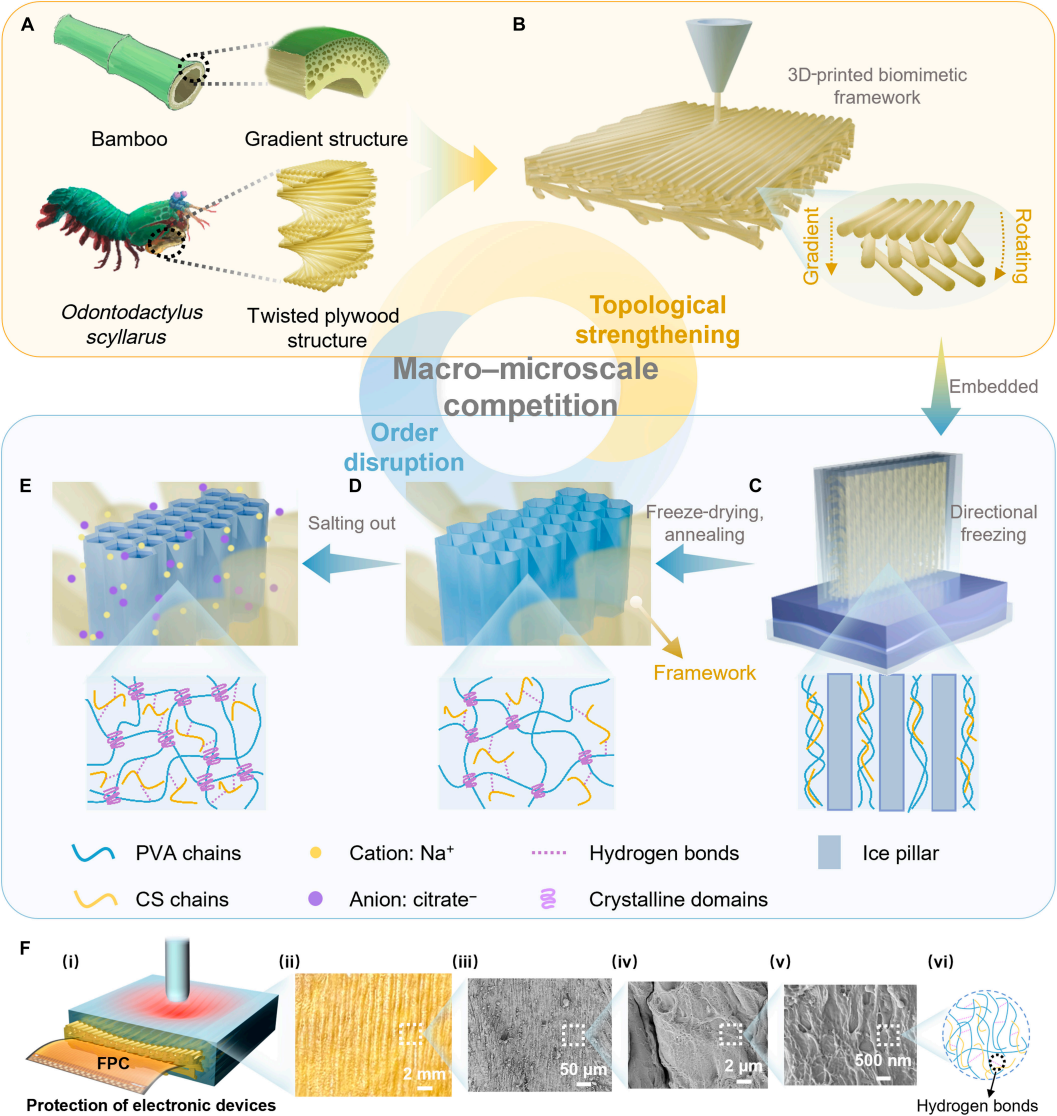
Supra-biomimetic design, fabrication process, and hierarchical structure of GT-HA composites. (A) Gradient structure of bamboo and twisted plywood structure of *Odontodactylus scyllarus*. (B) Biomimetic GT framework prepared via 3-dimensional (3D) printing. (C to E) Schematic illustrations of directional freeze-casting-induced self-assembly and annealing-assisted salting-out strategies for GT-HA composites. (F) Schematic illustration of GT-HA composites for impact protection of electronic devices (i) and its multiscale hierarchical structure (ii to vi). (ii) Macroscopic view; (iii to v) Scanning electron microscope (SEM) images of the micro- and nanostructures; (vi) schematic of polymer chain interactions. GT, gradient-twisted plywood; HA, hierarchically anisotropic.

Here, we propose a supra-biomimetic design strategy that addresses these limitations by embedding a precisely tailored, 3-dimensionally printed (3D-printed) gradient-twisted plywood (GT) framework into a hydrogel matrix, combined with synergistic molecular and structural engineering to fabricate a topologically controllable, hierarchically anisotropic (HA) hydrogel composite (denoted GT-HA composite). The GT framework is explicitly designed to harness and regulate the MMC mechanism: its gradient and spiral geometry coordinates macroscale stress guidance and crack deflection with micro/nanometer fiber sliding, nanocrystalline formation, and dynamic molecular interactions, thereby transforming competition into synergy. Quasi-static compression and dynamic impact experiments demonstrate that the GT-HA composite attenuates up to 88% of impact force under low-velocity impact and achieves a compressive strength of 183.57 MPa at strain rates up to 4,093 s^−1^, which is superior to those of many reported anti-impact soft materials. Computational modeling further reveals that the synergistic effect between the GT framework and the hierarchically anisotropic structure considerably enhances impact resistance and elucidates the energy-dissipation mechanism of GT-HA composites under impact. Moreover, due to its good compatibility with IC/MEMS processes, the GT-HA composite enables large-area integration with wafers, effectively protecting high-value devices from high-speed impact damage (Fig. 1F (i)). This work not only bridges the gap between structural biomimetics and scalable manufacturing but also establishes a general principle, the deliberate regulation of macro–micro competition, for designing next-generation protective materials.

## Results

### Design and fabrication strategy of composites

As illustrated in Fig. 1A, inspired by the superior mechanical properties of natural biomaterials, we integrated the gradient structure into the twisted plywood structure and fabricated a precisely controllable biomimetic GT framework via 3D printing (Fig. 1B). Subsequently, this framework was embedded into a hydrogel to construct a topologically controllable supra-biomimetic HA hydrogel composite (GT-HA composite), by combining directional freeze-casting-induced self-assembly and annealing-assisted salting-out methods. Three-dimensional printing enables precise regulation of material deposition and macroscopic structural morphology due to its high controllability and precision down to 10 μm, thus achieving accurate fabrication of gradient sizes and rotation angles to regulate the MMC. Furthermore, PVA is selected as the primary material for both the framework and the hydrogel matrix due to its stable, nontoxic nature, excellent hydrophilicity, and tunable crystallinity. This supra-biomimetic design considerably enhances the integration between the GT framework and the hydrogel matrix, resulting in a robust interface that spans from mechanical interlocking to molecular-level fusion. Chitosan (CS), serving as an auxiliary material for the hydrogel, forms multiple hydrogen bonds with PVA to reconstruct the PVA network [42]. The specific processing for the GT-HA composite is illustrated in Fig. 1C to E. First, the GT framework was immersed in a PVA/CS polymer precursor solution and then subjected to directional freezing by contact with a liquid-nitrogen-cooled copper block. Driven by the temperature gradient between liquid nitrogen and the precursor solution, the solution and GT framework were firmly frozen into a single entity. This process simultaneously induced the phase separation of the solution to increase the concentration of the PVA phase and promote the entanglement of its long chains. Subsequently, hydrogen bonds formed between the concentrated PVA and hydroxyl groups on CS chains, ultimately forming a honeycomb-like polymer wall microstructure with directional pores, high amorphousness, and low crystallinity [35]. Afterward, freeze-drying was performed at −50 °C and 10 Pa for 48 h, during which ice crystals gradually sublimated from the hierarchical porous network of the hydrogel. The hydrogel was then annealed at 90 °C for 60 min to greatly enhance its crystallinity [43]. Finally, the annealed hydrogel was salted out in a saturated sodium citrate (Na_3_Cit) solution for 48 h. During this period, the polymer chains underwent intense aggregation and separated from the original homogeneous phase, further forming a reticular nanofiber network on the surface of the micrometer-scale aligned pore walls [38]. It ultimately yielded the supra-biomimetic GT-HA composite with a hierarchically anisotropic structure. As shown in Fig. 1F (ii), the GT-HA composite exhibits tight integration between the macroscopic GT framework and the hydrogel at the macroscopic scale. Further observations in Fig. 1F (iii) to (v) reveal obvious anisotropy at the micrometer and a nanometer aggregated network on the wall surface, indicating that the high aggregation and crystallization of the composite further strengthen the rigid polymer walls. The molecular chains and hydrogen bonds at the molecular level are schematically illustrated in Fig. 1F (vi).

To investigate the effects of the aforementioned supra-biomimetic design strategy on mechanical properties, we also prepared a series of control samples for comparison. Specifically, isotropic (I) and anisotropic (A) PVA/CS hydrogels were first fabricated via traditional freeze–thaw and directional freezing methods, respectively. Subsequently, the A hydrogel underwent salting-out treatment to yield anisotropically enhanced (AE) PVA/CS hydrogels. Furthermore, an HA PVA/CS hydrogel was developed by combining directional freeze-casting-induced self-assembly and annealing-assisted salting-out. Finally, P-HA and T-HA PVA/CS composites were constructed by introducing porous (P) and twisted plywood (T) frameworks into the HA hydrogel matrix, respectively. Both composites possess topological frameworks with dimensions and volume ratios identical to those of the GT framework (Fig. S1), which are designed to investigate the regulatory effect and underlying mechanisms of the framework on the mechanical properties of hydrogels. A representative rotation angle of 18° was selected to balance the sample thickness and the integrity of the helical period [44,45] (see Materials and Methods for more details).

### Microstructure and quasi-static mechanical properties

**Fig. 2.**
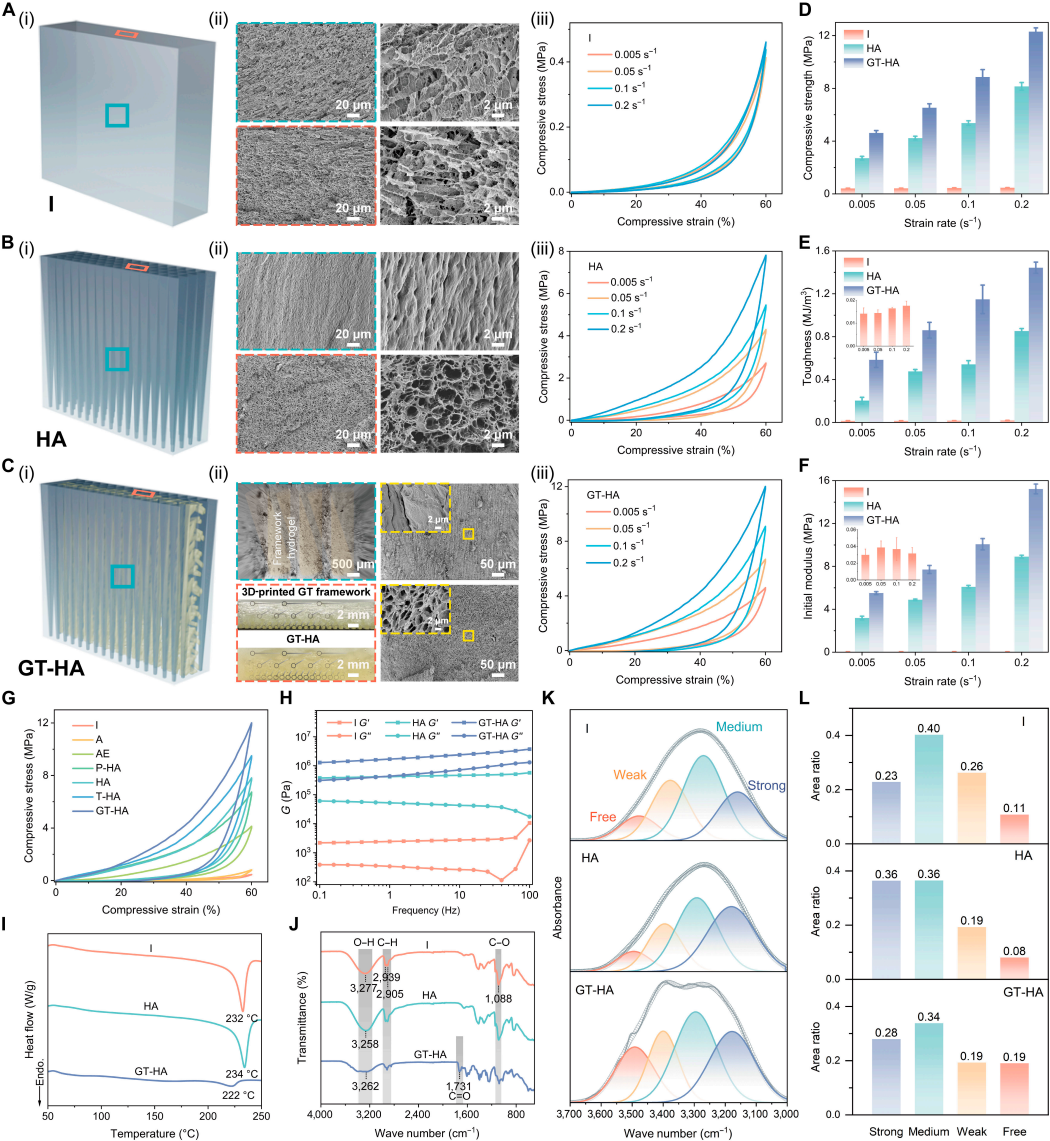
Morphology and mechanical property characterization of different samples. (A to C) Schematic illustrations (i), scanning electron microscope (SEM) images in different directions (ii), and compressive stress–strain curves at different strain rates (iii) of isotropic (I) hydrogels (A), HA hydrogels (B), and GT-HA composites (C). (D to F) Compressive strength, toughness, and initial modulus. Data are presented as mean ± standard deviation from 3 independent samples (*n* = 3). (G) Compressive stress–strain curves of different samples at a strain rate of 0.2 s^−1^. (H to J) Rheological (H), differential scanning calorimetry (DSC) (I), and Fourier transform infrared (FTIR) (J) results of different samples. (K and L) Peak-fitting curves of the FTIR spectra of different samples and the area ratios of strong, medium, and weak hydrogen bonds, as well as free hydroxyl groups. GT, gradient-twisted plywood; HA, hierarchically anisotropic.

To verify the positive effects of the GT framework and HA structure on the mechanical properties of GT-HA composites, we benchmarked all of the aforementioned samples for mechanical property analysis. Figure 2A to C compares the morphologies and compressive stress–strain curves at various strain rates of 3 representative sample groups: I hydrogels, HA hydrogels, and GT-HA composites. Figure 2A (ii) shows that the internal networks of I hydrogels exhibit uniform disorder and loose cross-linking, with random porous structures in all directions due to random ice crystal growth. This results in relatively low compressive strength, toughness, and modulus at different strain rates, as depicted in Fig. 2A (iii). Regarding the HA hydrogel, it successfully constructs a hierarchically anisotropic structure, as demonstrated in Fig. 2B (i) and (ii), through a combination of directional freeze-casting-induced self-assembly and annealing-assisted salting-out. The image in the blue dashed box shows a highly ordered PVA/CS structure aligned along the ice crystal growth direction, and the top view in the red dashed box confirms a porous honeycomb network formed by a 3-dimensionally interconnected PVA/CS structure. Compared with simpler anisotropic hydrogels (e.g., A and AE hydrogels; see Fig. S2), the HA hydrogel exhibits enhanced mechanical properties (Fig. 2B (iii)) via internally aligned micropore walls, densified nanofibers, and molecularly cross-linked PVA/CS chains. Its compressive strength (7.8 MPa) and toughness (0.86 MJ m^−3^) substantially exceed those of I, A, and AE hydrogels. By integrating a GT framework with the hierarchically anisotropic structure, the mechanical properties are further enhanced to outperform those of most previously reported hydrogel composites [46,47]. Figure 2C (ii) shows the 3D-printed GT framework tightly integrated with the hydrogel in the blue dashed box; the right-hand inset reveals a highly ordered structure aligned with the ice crystal growth direction, and the yellow dashed box further highlights a reticular nanofiber network formed on the surfaces of micrometer-scale aligned pore walls. The comparative image in the red dashed box confirms the successful embedding of the GT framework within the hydrogel, with the right-hand magnification showing a porous honeycomb network in the hydrogel matrix. Figure 2C (iii) indicates that incorporating the GT framework markedly enhances the mechanical properties of the composite, achieving compressive strength and toughness of 12 MPa and 1.4 MJ m^−3^, respectively. Furthermore, although the P-HA and T-HA composites also exhibit distinct morphological anisotropy (Fig. S3), their strength is markedly lower than that of the GT-HA composite (Fig. S4). A particularly intriguing observation is the compressive strength hierarchy. P-HA composites exhibit lower strength than the HA hydrogel without a framework, which in turn is weaker than the T-HA composites (Fig. 2G). This indicates that the introduction of a macroscopic framework does not invariably enhance the mechanical properties of the composites; instead, a competitive interplay exists between the topological reinforcement at the macroscale and the energy-dissipation mechanisms at the microscale. We term this phenomenon the MMC mechanism in supra-biomimetic composites, which is elaborated in the following sections. Owing to this competition, the topological architecture of the macroscopic framework must be deliberately designed and fabricated to enable effective synergy between the 2 scale-dependent mechanisms. The GT framework represents such a finely engineered architecture, and the superior performance of GT-HA once again demonstrates that rational utilization of this competition mechanism can lead to exceptional supra-biomimetic compressive performance [48]. Furthermore, we investigated the compressive properties of 3 representative samples at different strain rates. As shown in Fig. 2D to F, when the strain rate increases from 0.005 to 0.2 s^−1^, the GT-HA composite exhibits obvious strain hardening at high loading rates [49], and this strengthening effect is markedly more pronounced than that of I and HA hydrogels. The GT-HA composite has a strength of 4.6 MPa at 0.005 s^−1^ and 12 MPa at 0.2 s^−1^. Its energy-dissipation density, defined as the area of the stress–strain hysteresis curve, reaches 0.54 MJ m^−3^ at 0.005 s^−1^ and 1.4 MJ m^−3^ at 0.2 s^−1^. This performance reflects its potential for applications in impact resistance and energy absorption.

We characterized the rheological properties, thermal performance, and functional groups of different samples using rheological tests, differential scanning calorimetry (DSC), and Fourier transform infrared (FTIR) spectroscopy. The rheological tests (Fig. 2H) revealed that the I hydrogel exhibited the lowest storage modulus (*G*′), and its loss modulus (*G*″) first decreased and then increased at high frequencies, indicating a loosely packed network structure. With the sequential introduction of directional freezing, salting-out, and annealing processes, both the *G*′ and *G*″ of the hydrogels increased progressively from the A to the HA hydrogel, while the frequency dependence of *G*″ weakened (Fig. S5a). The ordered crystalline structures induced by these processes effectively enhanced network elasticity and facilitated more marked and stable viscous dissipation. Notably, the GT-HA composite exhibited the highest *G*′ and *G*″ alongside the flattest frequency response, providing direct mechanical evidence for its excellent elastic recovery and stable energy dissipation. DSC results (Fig. 2I and Fig. S5b) indicated that the melting temperature (*T*_m_) of the HA hydrogel increased to 234 °C, higher than that of the I hydrogel (232 °C), verifying that process optimization strengthened the ordered crystalline structure. However, the *T*_m_ of GT-HA composite decreased to 222 °C with reduced peak intensity, suggesting that the embedded GT framework disrupted the hydrogel matrix’s long-range order and crystallinity [50], which is a clear signature of the MMC. Crucially, despite this reduction in crystallinity [51], the GT-HA composite maintained mechanical performance superior to that of the HA hydrogel, demonstrating that the macroscopic topological reinforcement provided by the GT framework outweighs the sacrifice in microscale order, thereby converting the MMC from a conflict into a synergistic design advantage. FTIR spectroscopy (Fig. 2J and Fig. S5c) showed that the characteristic –OH peak of the hydrogels shifted from 3,277 cm^−1^ (I hydrogel) to 3,258 cm^−1^ (HA hydrogel), while the HA hydrogel exhibited enhanced intensities of the symmetric double peaks at 2,939 and 2,905 cm^−1^ as well as the peak at 1,088 cm^−1^, all of which indicated strengthened hydrogen bonding interactions [52]. Quantitative peak fitting (Fig. 2K and L and Table S1) further confirmed a marked increase in the area fraction of strong hydrogen bonds in the HA hydrogel than in the I hydrogel, indicating its highly ordered hydrogen-bond network. In the GT-HA composite, a high proportion of medium and strong hydrogen bonds was maintained, while the fraction of free hydroxyl groups increased to 0.19. This phenomenon originates from robust interfacial coupling between the GT framework and the hydrogel matrix: interfacial pinning confines the conformational motion of macromolecular chains, disrupts the long-range-ordered hydrogen-bond arrangement, and triggers the dissociation of free hydroxyl groups. This molecular-level structural evolution directly verifies the microscale degradation induced by the MMC mechanism. Additionally, the appearance of a characteristic C=O peak at 1,731 cm^−1^ in the GT-HA composite verified the successful integration of the framework.

### Dynamic impact performance

**Fig. 3.**
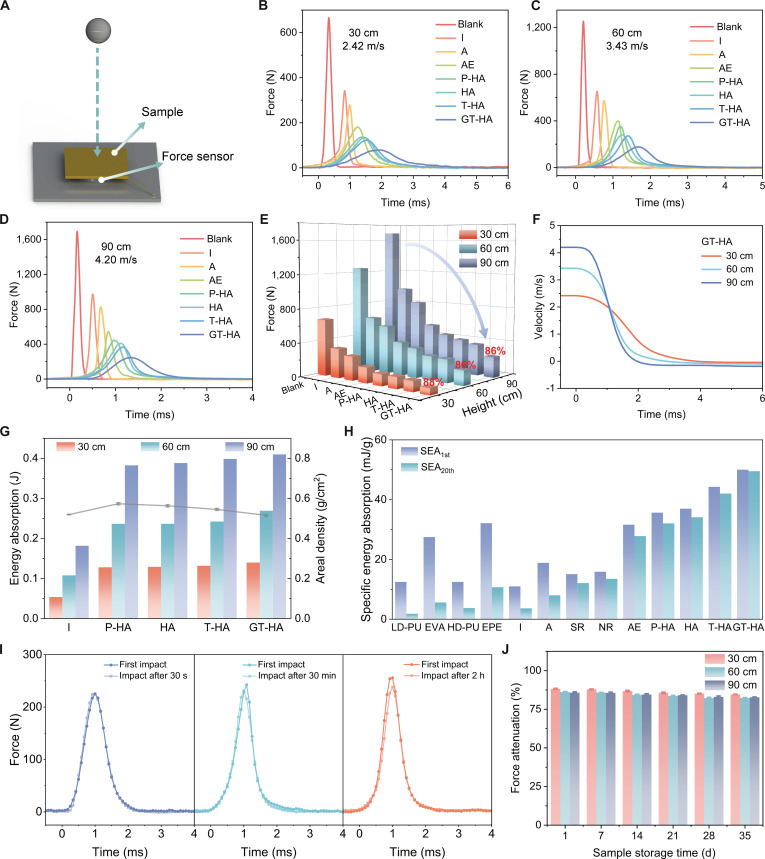
Low-velocity impact performance and stability of GT-HA composites. (A) Schematic illustration of the falling-ball impact system. (B to D) Force–time curves of different samples during impact at heights of 30 (B), 60 (C), and 90 cm (D). (E) Peak forces of different samples compiled from the 3 independent experiments above. (F) Velocity–time curves of GT-HA composites during impact. (G) Energy-absorption performance and areal densities of different samples at different heights. (H) Specific energy absorption (SEA) of different materials under the 1st and 20th drop-weight impacts. (I) Force–time curves of the GT-HA composite at different time intervals after drop-weight impact from 90 cm. (J) Stability of the impact attenuation capability of GT-HA composites over 35 d. GT, gradient-twisted plywood; HA, hierarchically anisotropic.

We performed low-velocity falling-ball impact tests to evaluate the impact resistance of various samples. As illustrated in Fig. 3A, a 55-g steel ball was released from different heights onto samples fixed over a force sensor. Effective impact resistance is reflected by a lower peak force and a longer buffer time. Force–time curves under varying impact heights are shown in Fig. 3B to D, while the summarized peak forces, energy-absorption performance, and areal density are presented in Fig. 3E and G. Evidently, as height increased from 30 to 90 cm, the blank group exhibited a sharp rise in peak force from 665.59 to 1,692.5 N, with buffer times below 1 ms, indicating high impact damage risk. I hydrogels dissipated only a portion of the impact energy under impact loading due to structural collapse, and the peak force decreased by only 49%, 48%, and 42% relative to that of the blank group as the height increased from 30 to 90 cm. At a 90-cm drop height, the energy absorption was only 0.18 J with an energy-dissipation efficiency of 37%. The A and AE hydrogels exhibited limited impact-protection performance due to the incorporation of directional freezing and salting out, with the peak force attenuated by 52% and 68%, respectively, at a height of 90 cm. In contrast, the HA hydrogel, featuring a hierarchically anisotropic structure, effectively resisted deformation and reduced damage under impact loading. It yielded peak forces of 133.31, 280.37, and 399.82 N at 3 heights, with peak-force attenuation of 80%, 78%, and 76% relative to that of the blank group, respectively. The buffering time extended to approximately 3, 2.8, and 2.5 ms, respectively, and the energy-dissipation efficiency reached 80%. These results highlight that finer ordering alone, while beneficial, cannot fully harness the dissipation potential across scales, as the lack of a macroscopic guiding framework leaves the system vulnerable to stress localization, a manifestation of the unregulated MMC. Introducing a topological framework does not always improve performance, however. Both the impact resistance and energy-absorption capacity gradually increase in the order of P-HA, HA, T-HA, and GT-HA, which follows the same trend observed in quasi-static compression tests. Under impact loading, P-HA undergoes rapid pore-wall buckling and fracture, enabling fast but inefficient energy absorption, whereas T-HA’s crack deflection and interfacial dissipation are partially suppressed due to time constraints. Both cases illustrate the MMC in action: a poorly matched macroscopic framework can disrupt, rather than synergize with, the finer-scale dissipation pathways. Notably, the GT-HA composite overcomes this competition. With the incorporation of the GT framework, peak forces drop to 78.31, 176.14, and 243.66 N at 30, 60, and 90 cm, respectively, corresponding to attenuations of 88%, 86%, and 86%, respectively, and buffering times are extended to ~4.2, 3.5, and 3 ms, respectively. At a 90-cm impact height, the energy absorption reaches 0.41 J with an efficiency of 85%. This stems from the regulation of the MMC through the GT framework and hierarchically anisotropic structure, which act across macro-to-molecular scales to distribute stress and dissipate energy at multiple levels. The detailed mechanism is elaborated in subsequent sections. Notably, GT-HA achieves the maximum energy absorption without increasing areal density and even exhibits superior areal density performance. This confirms that the multiscale energy-dissipation mechanism induced by the GT framework endows the material with protective performance far exceeding that of conventional composite designs. Figure 3F presents the velocity–time curves of the GT-HA composite at different heights, from which it can be observed that the residual velocity decreases to below 0 m/s. This indicates that the steel ball failed to penetrate the sample during impact and rebounded at all impact energy levels. By comparison, I, A, and AE hydrogels were fully penetrated with the residual velocity larger than 0 (Fig. S6), corroborated by postimpact scanning electron microscope (SEM) images (Fig. S7).

To eliminate the influence of sample size and mass, we compared the areal-density-normalized specific energy absorption (SEA) and force attenuation of different samples. GT-HA exhibits markedly higher SEA than commercial rubbers and porous foams, demonstrating that its superior impact resistance arises from the unique MMC mechanism rather than size or mass effects (Fig. S8a). Ultralight foams show high single-impact normalized performance due to low areal density (Fig. S8b), but their energy dissipation relies on catastrophic pore-wall collapse and irreversible plastic deformation, resulting in poor cyclic stability; after 5 impacts, force attenuation retention falls below 60% (Fig. S8c). In contrast, GT-HA retains 99% of its initial force attenuation and energy absorption after 20 consecutive impacts (Fig. 3H and Fig. S8d). Its SEA and SEA retention rate are markedly higher than those of common polymeric foams, including low- and high-density polyurethane (LD-PU and HD-PU), ethylene-vinyl acetate (EVA), and expandable polyethylene (EPE), and even surpass those of high-performance elastomers such as natural rubber (NR), silicone rubber (SR), and expanded polypropylene (EPP). SEM observations reveal only minor damage and a peak-force reduction of 2.5% (Fig. S9), indicating that excellent structural integrity is well maintained. This demonstrates that GT-HA dissipates impact energy not by structural damage but via multiscale cooperative pathways induced by the GT framework, which evenly distributes stress and prevents failure from stress concentration, overcoming the inherent limitation of conventional lightweight foams that show high single-impact performance but poor cyclic durability. Subsequently, time-dependent recovery tests were performed at 30 s, 30 min, and 2 h after impact. The results show that the peak impact force recovered to 99.66%, 99.27%, and 99.50% of its initial value, respectively, while the corresponding energy absorption recovered to 101.61%, 98.72%, and 98.69% (Fig. 3I). All recovery ratios approached 100% (Fig. S10), confirming that the transient load from a single falling-ball impact did not induce irreversible damage to the internal network. This behavior is primarily attributed to the synergistic interaction between the GT framework and the hydrogel matrix, which enables stable protective performance without obvious long-term relaxation or structural reconstruction. Repeated cyclic impact and time-dependent recovery tests further verify the excellent structural stability and fatigue resistance of this composite system under complex impact loading conditions.

Figure 3J reveals that after 35 d of immersion, the peak-force attenuation ratio of the GT-HA composite remains below 5% at all impact heights, and the material maintains structural integrity in aqueous environments (Fig. S11). Key physical parameters including mass loss, volume variation, water content, and the leaching of CS and PVA were quantitatively measured over 7 consecutive days of immersion, further verifying the excellent long-term stability of the composite (Fig. S12).

**Fig. 4.**
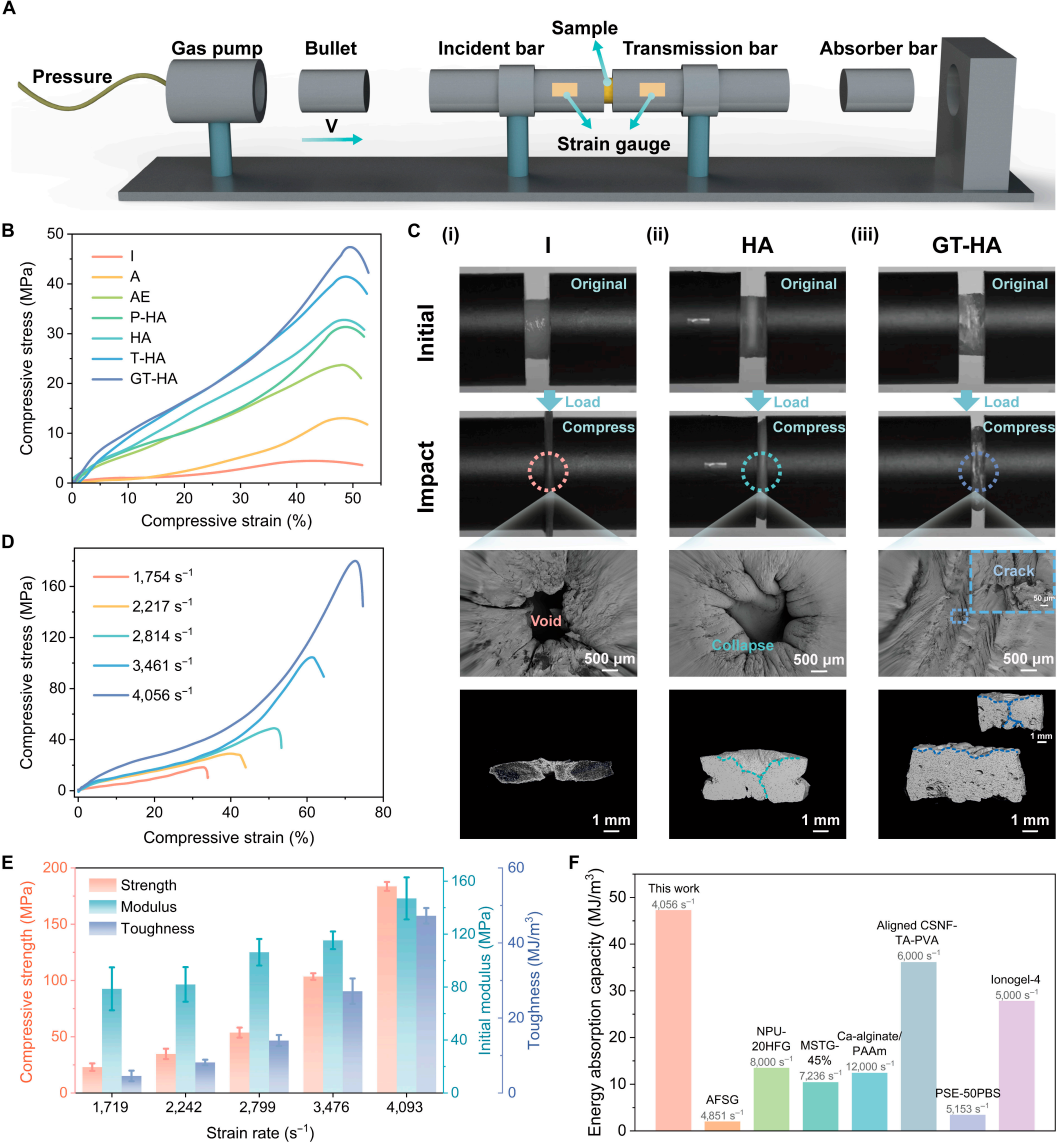
High-speed impact performance of GT-HA composites. (A) Schematic illustration of the split Hopkinson pressure bar (SHPB) testing system. (B) Compressive stress–strain curves of different samples during impact, with a strain rate of approximately 2,800 s^−1^. (C) Photographs of isotropic (I) hydrogels (i), HA hydrogels (ii), and GT-HA composites (iii) before and after impact, as well as scanning electron microscope (SEM) and micro-computed tomography (micro-CT) images after high-speed impact (strain rate: 2,800 s^−1^). (D) Compressive stress–strain curves of GT-HA composites at different strain rates, with data presented as mean ± SD (*n* = 3). (E) Relationships between the compressive strength, initial modulus, and toughness of GT-HA composites and strain rate. (F) Comparison of energy absorption between the GT-HA composite and reported impact-resistant soft materials at different strain rates. GT, gradient-twisted plywood; HA, hierarchically anisotropic.

We further characterized the high-strain-rate mechanical properties of the material via a split Hopkinson pressure bar (SHPB) and verified dynamic stress equilibrium by comparing the waveforms (Fig. S13), as shown in Fig. 4A. Under dynamic compression at a large strain rate of 2,800 s^−1^, the compressive strength increases from 4.45 to 47.4 MPa, with a sequence of I < A < AE < P-HA < HA < T-HA < GT-HA, as depicted in Fig. 4B. This stepwise improvement again reflects the progressive regulation of the MMC: the P-HA composite suffers deep pores due to wave-impedance mismatch and local stress concentration, and the T-HA composite shows shallower cracks owing to interlayer impedance gradients that promote stress-wave reflection and crack deflection (Fig. S14), whereas the GT-HA composite, by harmonizing macrotopological guidance with microscale dissipation, minimizes damage accumulation. Postimpact structural analysis at 2,800 s^−1^, including SEM and micro-computed tomography (micro-CT) images, reveals that the I hydrogel underwent catastrophic failure under impact, with its central region completely penetrated, as shown in Fig. 4C (i). Although the HA hydrogel exhibited improved strength, it still suffered deep cracks due to straightforward stress-wave propagation (Fig. 4C (ii)). In contrast, the GT-HA composite in Fig. 4C (iii) displays only shallow surface cracks, a direct consequence of its tortuous, multiscale dissipation path that slows crack penetration (Fig. S14). High-speed imaging (Movie S1) visually corroborates the sequential, noncatastrophic failure of the GT-HA composite compared to the abrupt fragmentation of I and HA hydrogels. Even at 3,500 s^−1^, where surface cracking becomes more apparent, the synergistic macro–micro interaction continues to deflect and dissipate energy effectively (upper right micro-CT image in Fig. 4C (iii) and Fig. S15). High-strain-rate compression tests across a wider rate range demonstrate pronounced strain-rate sensitivity. As depicted in Fig. 4D, the initial modulus and compressive strength of GT-HA composite rise markedly from 78.67 and 23 MPa at 1,719 s^−1^ to 147 and 183.57 MPa at 4,093 s^−1^ (Fig. 4E) [53]. As shown in Fig. 4F, Fig. S16, and Table S2, GT-HA achieves the highest energy-absorption capacity at the lowest strain rate, which fully demonstrates its excellent dynamic energy-dissipation efficiency among biomimetic impact-resistant hydrogel composites [54–60]. In conclusion, by harnessing the MMC, the GT-HA composite exhibits exceptional mechanical properties under dynamic loading, characterized by high force attenuation, high modulus, high toughness, and high strength (Fig. S17).

### Impact resistance mechanism

**Fig. 5.**
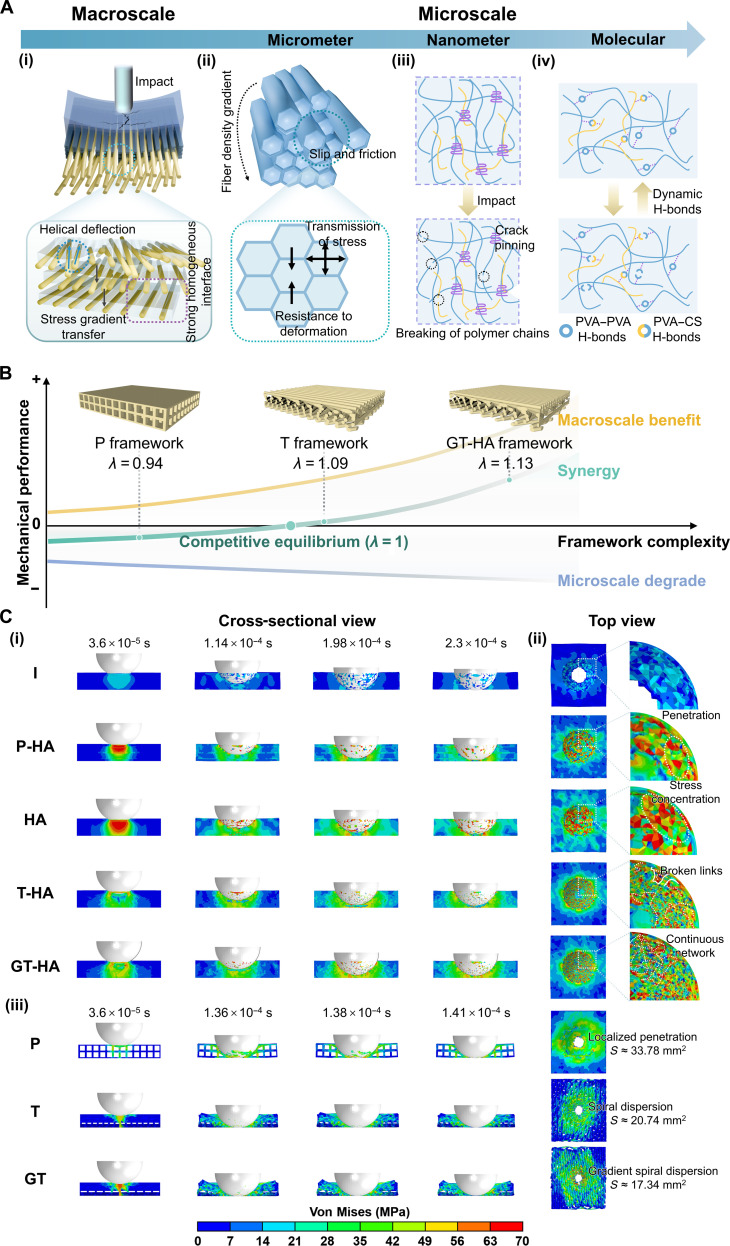
Impact resistance mechanism of GT-HA composites. (A) Multiscale impact resistance mechanisms of GT-HA composites. (i) Schematic illustration of impact resistance at the macroscale; (ii) density gradient of microscale fibers, fiber sliding and friction mechanisms, and interaction between microfiber walls and the internal matrix; (iii) polymer chain fracture and nanocrystalline domain formation under impact loading; (iv) dynamic molecular interactions. (B) The deliberate regulation of the macro–microscale competition (MMC) mechanism. (C) Stress distribution simulations of different samples during high-speed impact. (i) Cross-sectional views show the impact process of different samples; (ii) top views show the stress distribution of different samples at 0.23 ms after impact; (iii) cross-sectional view of the bare frame under impact and its top view at the instant of peak force (*S*, penetration area). GT, gradient-twisted plywood; HA, hierarchically anisotropic.

The GT-HA composite integrates high strength, toughness, and impact resistance through synergistic interaction between its GT framework and hierarchically anisotropic structure, enabled by successful regulation of the MMC mechanism. As illustrated in Fig. 5A (i), the macroscale GT framework guides and disperses impact stress via 2 complementary designs: a gradient structure with linearly varying layer widths creates a continuous stiffness gradient, directing stress waves from high- to low-modulus regions to achieve graded energy release and layered dissipation; a twisted plywood structure, with rotationally aligned adjacent layers, actively induces multi-level crack deflection and bifurcation, extending the failure path and enabling zonal energy absorption. At the micrometer scale, as illustrated in Fig. 5A (ii), fiber sliding and friction dissipate energy, and a 3D interwoven honeycomb fiber network confines deformation and redistributes stress via pore wall and matrix interactions, retaining elasticity and strength. The gradient layer thickness further spatially tunes energy dissipation by varying fiber density, a design that harmonizes the macroscopic stress field with microscopic frictional events. At the nanometer scale, polymer chain disentanglement and re-entanglement promote nanocrystalline aggregation and high crystallinity (Fig. S18) [61,62], which hinder crack propagation and enhance stress dispersion (Fig. 5A (iii)). Molecularly, energy is absorbed through the reversible breaking of hydrogen bonds among PVA and CS chains (Fig. 5A (iv)). Together, these macroscale and finer-scale interactions collectively form an integrated, sequential dissipation cascade.

It is important to emphasize that the MMC mechanism plays a decisive role in governing the impact resistance of these composites. As illustrated in Fig. 5B, while the introduction of a macroscopic framework can enhance mechanical performance through macroscale crack deflection and bifurcation (indicated by the macroscale benefit curve, yellow), it simultaneously disrupts the structural order of the hydrogel matrix at the microscale, leading to a microscale degradation (blue curve). The overall performance of the composite is thus determined by the competition between these 2 opposing effects. Notably, as the topological complexity of the macroscopic framework increases, the macroscale benefit grows considerably, whereas the microscale degradation remains relatively stable. This is because the macroscopic framework and the microstructure differ by several orders of magnitude in scale; changes in macroscopic topology do not induce substantial variations in microscale order. Figure 5B also clearly demonstrates that not all of the macroscopic framework enhances the composite’s mechanical properties, with overall performance improving only beyond a competitive equilibrium point, a direct consequence of the MMC. Both our quasi-static and dynamic experiments consistently show the performance ranking P-HA < HA < T-HA. This trend conclusively validates the existence of the MMC and underscores the critical importance of tailoring the macroscopic framework’s topology. The GT framework presented here is precisely such a deliberately designed and optimized architecture, engineered to maximize the macroscale benefit, thereby transforming competition into synergy and achieving unprecedented impact resistance. By contrast, P-HA and T-HA composites lack the macroscale GT architecture and thus fail to regulate the MMC effectively. The P framework concentrates stress and disrupts microscopic order, while the T framework achieves crack deflection but does not fully integrate gradient stress guidance. Consequently, both exhibit inferior mechanical performance. Notably, based on polymeric viscoelastic network theory and heterogeneous composite mechanics, we deduce that the energy-dissipation mechanisms of the composite exhibit a pronounced strain-rate dependence. At low rates, energy dissipation is governed by molecular/microscale dynamics of chain and hydrogen-bond reorganization; at high rates, the macroscopic topological mechanisms dominate, managing inertial and stress-wave effects through graded stiffness and spiral-induced crack deflection. To quantitatively characterize the MMC mechanism, we introduce a synergy factor *λ*, defined as the ratio of the composite’s actual energy absorption to the sum of the energies of the isolated framework and hydrogel (*λ* = *E*_composite_/(*E*_framework_ + *E*_hydrogel_)), where the denominator represents the maximal theoretical nonsynergistic reference. A critical value of *λ* = 1 indicates the transition between competition-dominated and synergy-dominated states: *λ* < 1 (e.g., for the P-HA composite, *λ* = 0.94) reflects predominance of microscale degradation, whereas *λ* > 1 (e.g., for the GT-HA composite, *λ* = 1.13) confirms that macroscopic mechanical gain successfully overcomes microlevel weakening, achieving true synergistic enhancement (Table S3). All calculations were performed under identical geometric and boundary conditions, using the full-size pure hydrogel as a conservative reference. Sensitivity analyses of mesh discretization and interfacial friction indicate that *λ* remains strictly greater than 1 under all reasonable perturbations, with relative deviations below 3% (Fig. S19 and Tables S4 and S5), demonstrating the robustness of this quantitative criterion. Moreover, *λ* increases monotonically with the topological complexity of the framework, indicating that macroscopic gains progressively dominate, driving the system from a competitive state toward synergy.

Following mesh convergence analysis and experimental validation (Fig. S20), falling-ball impact finite element (FE) simulations were conducted to further compare the energy-dissipation behavior of various samples. As shown in Fig. 5C (i) and (ii), the I hydrogel is fully penetrated, indicating poor protection (Fig. S21). Both P-HA and HA samples exhibit localized stress concentration (Fig. S21). Although the incorporation of the P framework reduces stress in some regions (with noticeably larger blue regions than in the HA hydrogel), the P framework itself undergoes severe localized penetration (penetration area *S*: 33.78 mm^2^; Fig. 5C (iii)). The resulting macroscopic benefit is insufficient to offset the microscale degradation in the composite, resulting in localized perforation (Fig. S21), thus providing inferior protection compared to the HA hydrogel. In contrast, fine-tuning the MMC in T-HA and GT-HA composites effectively channels stress and greatly mitigates stress concentration. Although the T-HA composite displays a relatively uniform stress distribution, numerous fractured chains and interrupted load-transfer paths still exist within the system. This corresponds to the mere spiral stress dispersion effect of the T framework (*S*: 20.74 mm^2^), allowing only preliminary macro–microscopic synergy in T-HA composites (Fig. 5C (iii)). By comparison, benefiting from the GT framework, the GT-HA composite achieves more uniform stress distribution and more efficient energy dissipation (Fig. 5C (iii)) with a penetration area of only 17.34 mm^2^, which strengthens the macro–microscopic coupling and weaves all stress into a continuous network (Fig. 5C (ii) and Fig. S21) and thus promotes more effective stress transfer while driving crack deflection toward the hydrogel matrix. These simulation results align well with the experimental data in Fig. 4B to D, confirming the superior impact resistance and noncatastrophic failure behavior of the GT-HA composite, a direct outcome of the rationally regulated MMC mechanism.

### Application demonstration of composites in impact resistance

**Fig. 6.**
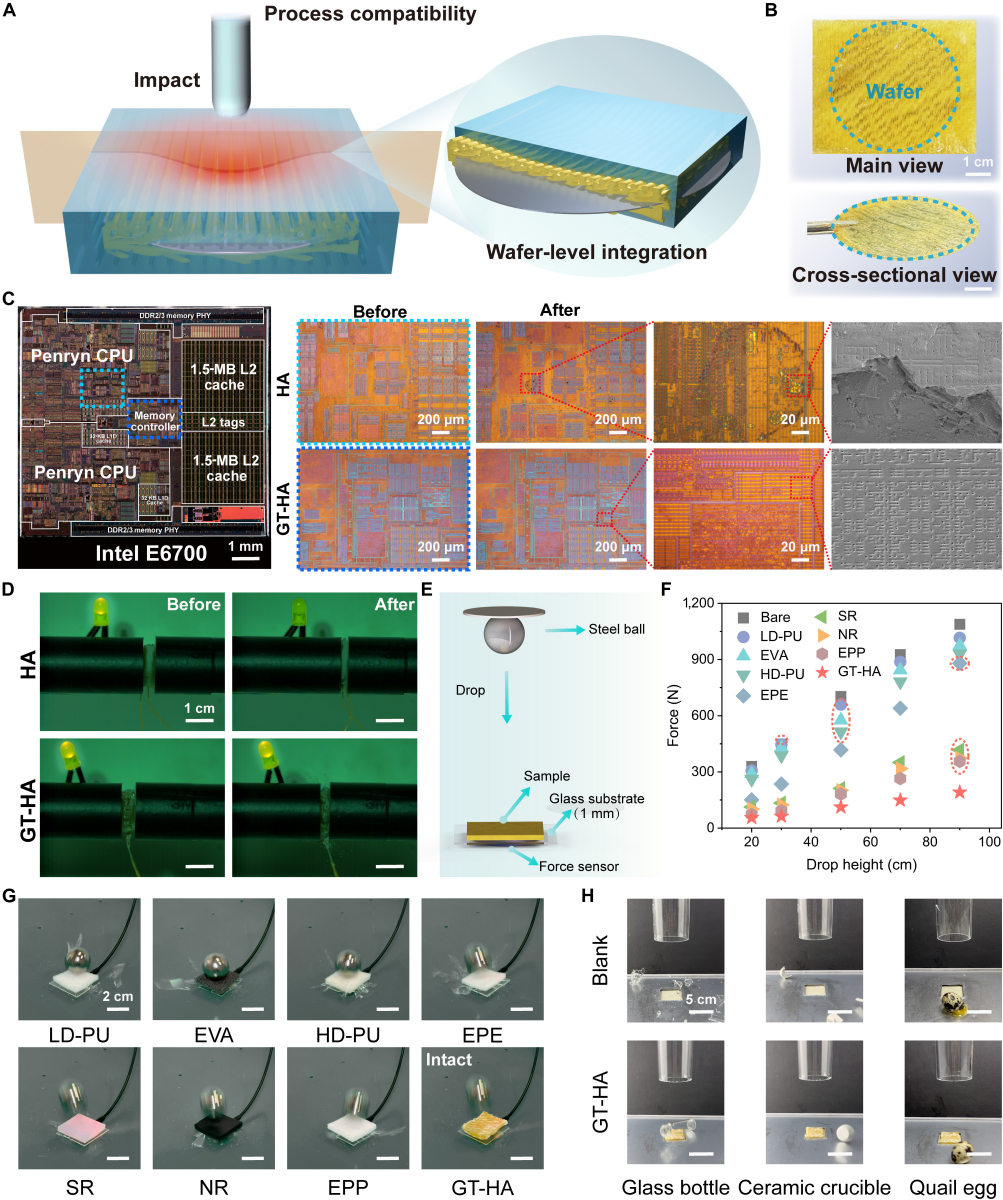
Impact-protection applications of GT-HA composites. (A) The fabrication process of GT-HA composites is compatible with integrated circuit (IC)/microelectromechanical system (MEMS) technologies. (B) Main and cross-sectional views of the GT-HA composite embedded with wafers. (C) The microstructure of the Intel E6700 processor die; optical micrographs and magnified scanning electron microscope (SEM) images of the HA hydrogel and GT-HA composite embedded with the processor die before and after high-speed impact. (D) Photographs of the HA hydrogel and the GT-HA composite embedded with flexible printed circuits (FPCs) before and after high-speed impact. (E) Schematic illustration of impact-protection performance. (F) Peak impact forces of different samples for glass protection at heights ranging from 20 to 90 cm (red circles indicate the initial height at which glass breakage occurs). (G) Impact snapshots of different samples for glass protection (*H* = 90 cm). (H) Photographs of glass bottles, ceramic crucibles, and quail eggs freely dropped from a height of 1.5 m onto the floor and the surface of the GT-HA composite. GT, gradient-twisted plywood; HA, hierarchically anisotropic.

Electronic devices, as core components of modern technology, require effective impact protection to ensure reliability and service life. The fabrication process of our GT-HA composite is compatible with standard IC/MEMS technologies, allowing wafer-level chip integration within the hydrogel matrix, as illustrated in Fig. 6A and B. This approach addresses long-standing challenges in scalable manufacturing of heterogeneous material systems while preserving both electronic and composite functionalities under dynamic mechanical impact (Fig. S22).

To evaluate the protective performance, we integrated an Intel E6700 processor die (65-nm production node) and a flexible printed circuit (FPC) into the GT-HA composite using a compatible process. Samples were subjected to impact tests via an SHPB at a strain rate of 2,000 s^−1^. As shown in Fig. 6C, postimpact SEM imaging reveals severe mechanical damage to the die embedded in pure HA hydrogel, whereas the on-die transistors and metal interconnects inside the GT-HA composite remain intact, demonstrating effective impact protection (Movie S2). Furthermore, during SHPB testing, a light-emitting diode (LED) connected to the FPC embedded in HA hydrogel extinguished upon impact, suggesting solder joint failure, while the LED linked to the GT-HA composite-embedded FPC remained illuminated (Fig. 6D and Movie S2). These results confirm the structural and functional integrity of the FPC protected by the GT-HA composite, underscoring its superior impact resistance in practical electronic packaging scenarios.

The exceptional energy absorption and force attenuation capabilities of the GT-HA composite translate into other impact resistance scenarios. To evaluate its performance in glass protection, we compared the composite against conventional cushioning foams (LD-PU, HD-PU, EVA, EPE, and EPP), NR, and SR. A 55-g steel ball was dropped from various heights onto glass slides covered with either reference materials or the GT-HA composite (35 × 35 × 5 mm^3^; Fig. 6E). As shown in Fig. 6F, the critical height for glass breakage (marked in red) increased gradually across bare, LD-PU, EVA, HD-PU, EPE, SR, NR, and EPP samples, reflecting progressively better impact resistance (Fig. S23). The critical fracture load of bare glass was 0.45 kN (*H* = 30 cm), whereas under the same conditions, GT-HA transmitted a peak load of only 0.06 kN (Fig. S24). When the drop height was increased to 90 cm, all conventional materials failed to prevent glass fracture (Fig. 6G), indicating limited energy dissipation under high-velocity impact. In contrast, GT-HA substantially attenuated the impact load, with a peak force of only 0.19 kN, far below the glass fracture threshold. This demonstrates its ability to effectively resist impact and suppress glass breakage, showing marked superiority over conventional materials (Movie S3). To further visualize the protective performance, a glass bottle, a ceramic crucible, and a quail egg were dropped from 1.5 m onto both a bare floor and a GT-HA composite. As captured in Fig. 6H and Movie S4, all items survived intact upon impact with the composite, in sharp contrast to the severe structural damage observed from bare floor impact. These results confirm the efficacy of the GT-HA composite for protecting fragile objects. Combining robust impact resistance with scalability, this material represents a promising alternative to commercial cushioning foams in product transport and protection.

## Discussion

In summary, we have developed a supra-biomimetic GT-HA composite that synergistically combines a 3D-printed gradient-twisted framework with an HA hydrogel matrix. Central to this design is the recognition and regulation of the MMC mechanism: the GT framework does not merely add a reinforcement but actively orchestrates stress transfer and crack deflection at the macroscale while synchronizing micrometer fiber sliding, nanometer nanocrystalline formation, and molecular-scale hydrogen-bond dynamics. This coordinated multiscale activation leads to unprecedented impact resistance, achieving up to 88% force attenuation under low-velocity impact and excellent dynamic strength and toughness. Additionally, the fabrication process is fully compatible with IC/MEMS processes, enabling wafer-scale integration that reliably protects high-value electronics such as processor dies and FPCs under high-speed impact. Beyond establishing a new benchmark for impact-resistant hydrogels, this work demonstrates a scalable material strategy that transforms inherent multiscale competition into a design principle, effectively merging biomimetic design with wafer-level manufacturing. We anticipate that this approach will enable advanced protection systems in areas such as portable electronics, autonomous devices, and aerospace instrumentation operating under extreme mechanical conditions.

## Materials and Methods

### Materials

PVA powder (molecular weight 85 to 124 kDa, 99% hydrolyzed) and Na_3_Cit (98%) were purchased from Aladdin Chemical Co., Ltd. (Shanghai, China). CS (molecular weight 300 kDa, degree of deacetylation 85%) was obtained from Shanghai Easen Chemical Technology Co., Ltd. (Shanghai, China). All chemical reagents were used as received without further purification unless otherwise stated.

### Preparation of hydrogels

#### Freeze–thawed hydrogel (I hydrogel)

A PVA/CS precursor solution was prepared by dissolving 10 wt% PVA and 0.5 wt% CS in deionized water under stirring at 95 °C for 2 h. After degassing, the solution was transferred into a polytetrafluoroethylene (PTFE) mold (3.5 mm [*L*] × 3.5 mm [*W*] × 5 mm [*T*]), frozen at −20 °C for 10 h, and then thawed at room temperature for 2 h. This freeze–thaw cycle was repeated twice. Afterward, the final sample was soaked in deionized water until equilibrium to obtain the I hydrogel.

#### Directional freeze-casting PVA/CS hydrogels

The same precursor solution was transferred into a copper-bottomed PTFE mold placed on a copper billet half-immersed in liquid nitrogen. During freezing, ice columns grew upward along the temperature gradient generated by the copper billet. The frozen sample was freeze-dried at −50 °C and 10 Pa for 48 h to sublime the ice, and the obtained aerogel was hydrated to yield the A hydrogel. For the AE hydrogel, the A aerogel was immersed in saturated Na_3_Cit solution for 48 h and then hydrated for 24 h. To prepare the HA hydrogel, the A aerogel was first annealed at 90 °C for 60 min and then subjected to the same salting-out treatment (48 h in saturated Na_3_Cit), followed by washing in deionized water for 24 h to remove excess ions.

#### GT-HA composite

The GT-HA composite was constructed by embedding a 3D-printed GT framework into a PVA/CS precursor solution. The GT framework was prepared via fused deposition modeling using a PVA filament. The filament was dried at 50 °C for 4 to 6 h and then deposited onto a heated bed coated with a PVA-specific adhesive (Shining 3D) using a commercial 3D printer (Bambu Lab X1 Carbon) following a computer-aided design model. After natural cooling, the framework was embedded into the precursor solution. The mixture was then transferred into a copper-bottomed PTFE mold on the liquid-nitrogen-cooled copper billet, frozen directionally, freeze-dried (48 h), annealed (90 °C, 60 min), and finally salted out in saturated Na_3_Cit for 48 h to yield the supra-biomimetic GT-HA composite.

### Characterization

The morphology and microstructure of the samples were observed using an SEM (Zeiss Supra 40). Chemical functional groups were analyzed by FTIR spectroscopy (Nicolet iS50, Thermo Nicolet, USA), with a wave number range of 4,000 to 400 cm^−1^. The thermal performance of the samples was obtained using a differential scanning calorimeter (DSC 214/TG 209 F3, NETZSCH, Germany). The samples were heated from 50 to 250 °C in a nitrogen atmosphere at a heating rate of 20 °C min^−1^. The rheological behavior of the samples was investigated using an Anton Paar MCR 302e modular advanced rheometer. Postimpact morphology and crack paths were visualized by micro-CT (Scanco vivaCT 80).

Quasi-static compression tests were conducted on a universal testing machine (INSTRON 3344, USA). All compression samples were cuboids (10 mm × 10 mm × 4 mm). Low-velocity impact resistance tests were performed using a falling-ball impact tester (Hongshan Instruments, China). A 55-g steel ball was dropped from a specified height to impact the sample placed on the surface of a force sensor. The sample dimensions were 35 mm × 35 mm × 5 mm, and the impact force was recorded by the force sensor. Each group of samples was tested at least 5 times, and the results were presented as mean ± standard deviation.

High-strain-rate compression tests were carried out using an SHPB apparatus. The SHPB system employed consists of a striker, an incident bar, a transmission bar, an absorber bar, and a data acquisition system. The compressed-air-driven striker impacts the incident bar to generate elastic stress waves, which propagate through the specimen. The acquisition system records the incident, reflected, and transmitted strain pulses, which are then converted to material mechanical parameters using one-dimensional stress-wave theory. The bars are made of aluminum with a diameter of 18.12 mm, and the strain gauges have a gauge factor of *K* = 1.9.

The specimen’s dynamic stress *σ*(*t*), strain *ε*(*t*), and strain rate $\dot{\varepsilon }\left(t\right)$ were calculated using standard one-dimensional stress-wave formulas:

$$\sigma \left(t\right)=E_{0}\frac{A_{0}}{A}\varepsilon_{t}\left(t\right)$$ (1)

$$\varepsilon \left(t\right)=-\frac{2C_{0}}{L}\int_{0}^{t}\varepsilon_{r}\left(t\right)\mathrm{dt}$$ (2)

$$\dot{\varepsilon }\left(t\right)=-2\frac{C_{0}}{L}\varepsilon_{r}\left(t\right)$$ (3)

where *E*_0_ is the elastic modulus of the bars; *A* and *A*_0_ are the cross-sectional areas of the specimen and bar, respectively; *L* is the initial specimen thickness; *C*_0_ is the elastic wave speed in the bars; and *ε_r_*(*t*) and *ε_t_*(*t*) are the reflected and transmitted strain signals, respectively.

A modeling-clay pulse shaper is mounted to the incident bar end to prolong the rise time of incident pulses, realize constant-strain-rate loading, and accelerate dynamic stress equilibrium. The specimens were cylindrical with a diameter of 10 mm and a height of 5 mm (length-to-diameter ratio = 1:2). This design balances the stiffness of the biomimetic framework and the viscoelastic dissipation of the hydrogel matrix. The tests were unconfined, free axial compression with unconstrained lateral boundaries. Prior to testing, thin layers of Vaseline were applied evenly to both contact faces between the specimen and the incident/transmission bars to eliminate friction-induced nonuniform deformation and local pseudo-confinement, rendering frictional effects negligible. The impact process was captured using a high-speed camera (Phantom V711, USA).

FE models of the I hydrogel, HA hydrogel, and GT-HA composite were established using the commercial software SolidWorks. All models had dimensions of 37 mm × 42 mm × 8.57 mm. The GT framework (overall thickness = 6.6 mm) was divided into 8 layers with rotation angles of 0°, 18°, 36°, 54°, 72°, 90°, 108°, and 126°, and the spacing between individual columns increased linearly from 1.5 mm at the top layer to 5 mm at the bottom layer. FE simulations of the impact process were performed using the commercial software Abaqus. An anisotropic constitutive model was established based on engineering constants and a potential function to describe the anisotropic mechanical behavior of the hydrogel, while its plastic response was characterized using a shear-yield criterion combined with strain hardening. Equivalent elastoplastic parameters were separately calibrated for the 4.2 m/s low-velocity impact and 50 m/s ultrahigh-velocity impact conditions, with consideration of dynamic rheological hardening arising from microstructural evolution at ultrahigh strain rates, including polymer chain disentanglement, microscopic dehydration, and nanoaggregate reinforcement. To accurately simulate interfacial failure between the hydrogel matrix and the PVA framework, zero-thickness cohesive elements were introduced. Damage initiation was governed by the quadratic nominal stress criterion, while damage evolution was described using an energy-based linear stiffness degradation model combined with the Benzeggagh–Kenane mixed-mode fracture criterion, enabling effective reproduction of interfacial debonding, delamination, and stress release. For the boundary conditions, the bottom support plate was fully constrained in all degrees of freedom, whereas the impact indenter was allowed to translate only along the loading direction. Hard contact and a penalty-based friction algorithm were applied to the contact surfaces, with distinct friction coefficients assigned to the external surfaces and internal interfaces. The initial mesh/contact tolerance was also optimized to ensure numerical stability. After mesh convergence analysis, 0.8 mm was selected as the optimal mesh size for mesh-independent calculations. Under the 4.2 m/s impact condition, the simulated force–time curve agreed well with the experimental result, validating the reliability of the constitutive model, contact algorithm, and parameter calibration strategy and providing a reliable basis for the 50 m/s ultrahigh-velocity impact simulations.

## Data Availability

The data that support the findings of this study are available from the corresponding authors upon reasonable request.
