# Supplementary material for: Supra-biomimetic Impact-Resistant Composites via Harnessing Macro–Microscale Competition
Source: Research (Wash D C). 2026 Jul 10;9:1358. doi: 10.34133/research.1358 (PMC13351122; doi:10.34133/research.1358)
Supplement: Supplementary 1 — Figs. S1 to S24 Tables S1 to S5 Movies S1 to S4 [file research.1358.f1.zip › Supplementary materials-revised-.pdf]

Supplementary Materials for

Supra-Biomimetic Impact-Resistant Composites via Harnessing Macro-Microscale Competition

Miao Lei[1,2], Mengqi Sun[3], Qixuan Zhu[1,3], Zihan Hao[1], Dehua Tan[1,2], Chaohui Wu[1,2], Yueying Yang[1], Chaohong Zhang[4], Xuewen Wang[1], Wei Huang[1,2,4]*, and Qianbo Lu[1,2]*

[1]State Key Laboratory of Flexible Electronics (LoFE) and Institute of Flexible Electronics (IFE), MIIT Key Laboratory of Flexible Electronics (KLoFE), Shaanxi Key Laboratory of Flexible Electronics, Northwestern Polytechnical University, Xi'an, China.

[2]Key Laboratory of Flexible Electronics of Zhejiang Province, Ningbo Institute of Northwestern Polytechnical University, Ningbo, China.

[3]School of Automation, Northwestern Polytechnical University, 127 West Youyi Road, Beilin District, Xi'an, China.

[4]School of Flexible Electronics and State Key Laboratory of Optoelectronic Materials and Technologies, Sun Yat-sen University, Shenzhen, China.

This supplementary information file includes:

Supplementary Figures S1 to S24

Supplementary Table S1 to S5

Supplementary Movies S1 to S4

Supplementary Figures and Texts

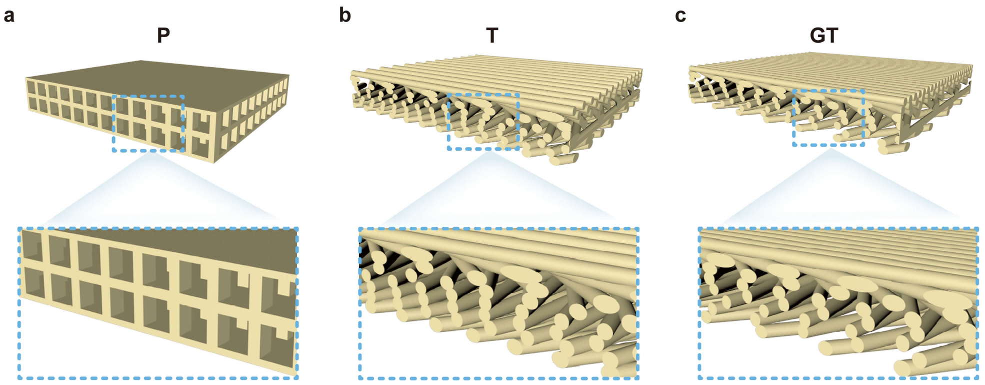

Fig. S1. Schematic illustrations of 3D-printed macroscopic frameworks. a-c, PVA frameworks with porous (P) structure (a), twisted plywood (T) structure (b), and gradient twisted plywood (GT) structure (c).

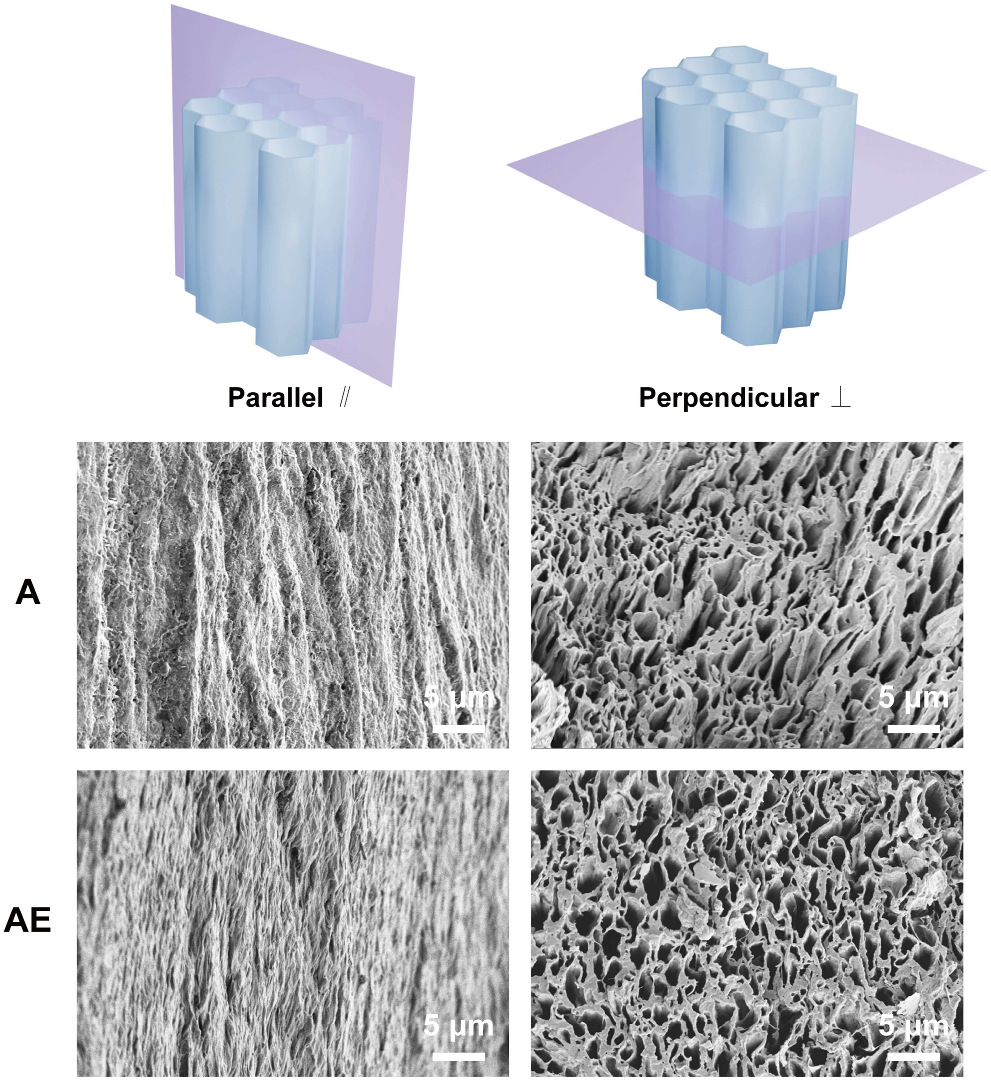

Fig. S2. Microstructures of hydrogel samples. Representative scanning electron microscopy (SEM) images of the hydrogel samples parallel (∥) and perpendicular (⊥) to the ice-growth direction for A

and AE hydrogels.

To investigate the regulatory effect of directional freeze-casting and salting-out on the microstructure of hydrogels, we observed the microstructure of A and AE hydrogels using SEM. Fig. S2 shows that both hydrogels exhibit distinct anisotropic characteristics: highly ordered fibers align parallel to the ice-growth direction, whereas relatively disordered porous structures are observed perpendicular to this direction. Further comparison reveals that the layered porous structure of AE hydrogels has a higher degree of densification than that of A hydrogels. This phenomenon indicates that directional freeze-casting can endow hydrogels with oriented pore wall structures, and salting-out can further strengthen the microstructure to induce stronger aggregation and crystallization behavior of polymer molecules.

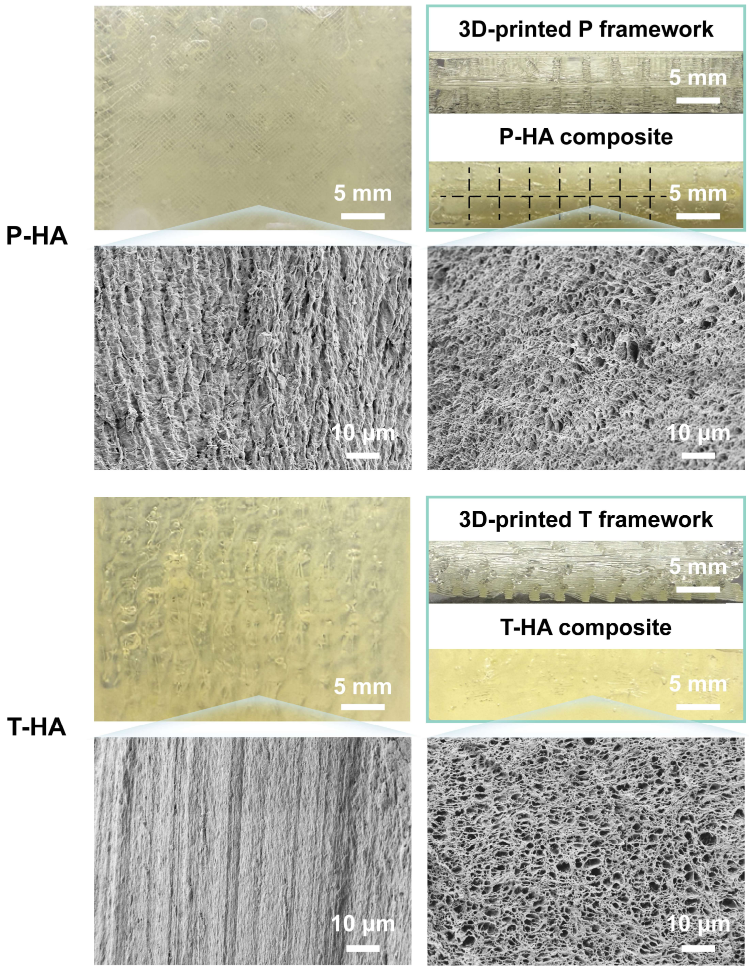

Fig. S3. Macroscopic photographs and SEM images of P-HA and T-HA composites parallel (∥) and perpendicular (⊥) to the ice growth direction.

Fig. S3 shows the macroscopic and microscopic structures of P-HA and T-HA composites, from which it can be observed that both composites exhibit significant anisotropic characteristics. Parallel to the ice-growth direction, both composites exhibit

ordered structural alignment, whereas perpendicular to it, comparison diagrams reveal tight bonding between the 3D-printed biomimetic topological frameworks and the hydrogel. Moreover, high-magnification images further demonstrate the porous honeycomb network inside the hydrogel. Notably, the P-HA composite exhibits lower structural order than the T-HA composite, likely because the P framework possesses inferior hierarchical complexity and spatial precision, which consequently disrupts the ordered structure of the hydrogel to a greater extent.

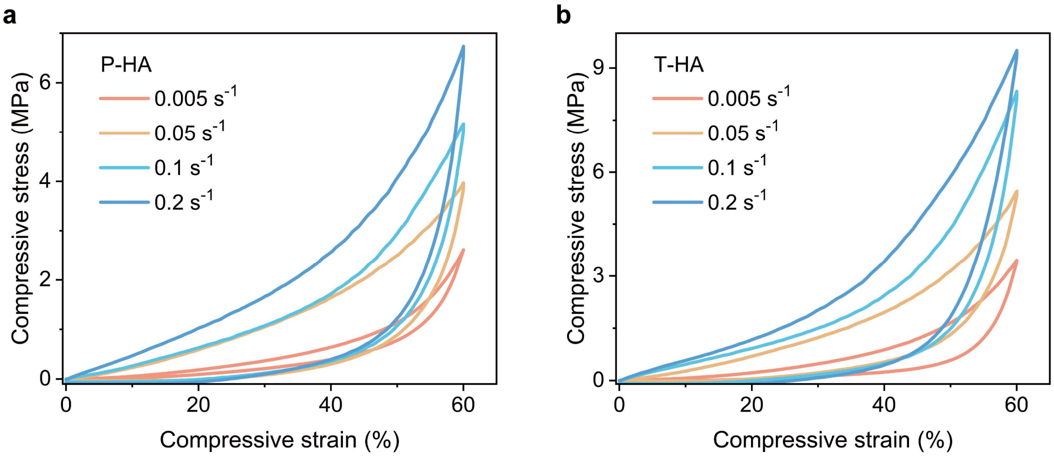

Fig. S4. Compressive stress-strain curves of P-HA (a) and T-HA (b) composites at different strain rates.

The Compressive stress-strain curves of P-HA and T-HA composites at different strain rates (Fig. S4) reveal obvious strain hardening behavior in both composites as the strain rate increases from 0.005 s^{-1} to 0.2 s^{-1}. However, T-HA composites display a more pronounced strengthening effect than P-HA composites. Specifically, at strain rates of 0.005 s^{-1} and 0.2 s^{-1}, the compressive strengths of P-HA are 2.61 MPa and 6.73 MPa, respectively, while those of T-HA reach 3.46 MPa and 9.50 MPa. This performance difference is consistent with the microstructural characteristics of the two composites in Fig. S3.

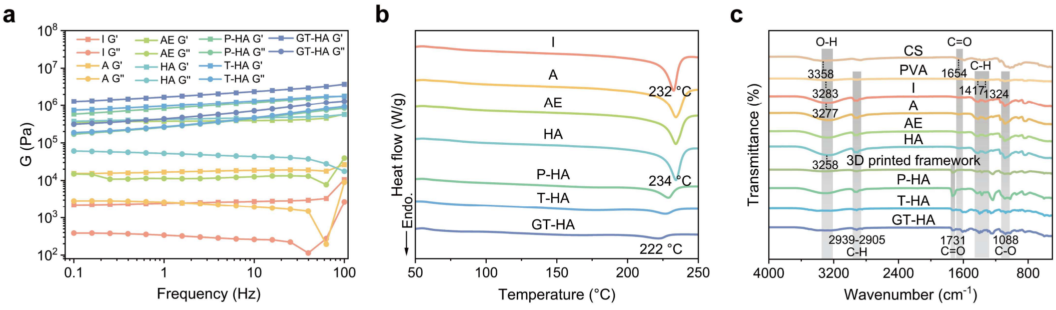

Fig. S5. Rheological test, differential scanning calorimetry (DSC), and Fourier transform infrared (FTIR) spectroscopy results of different samples.

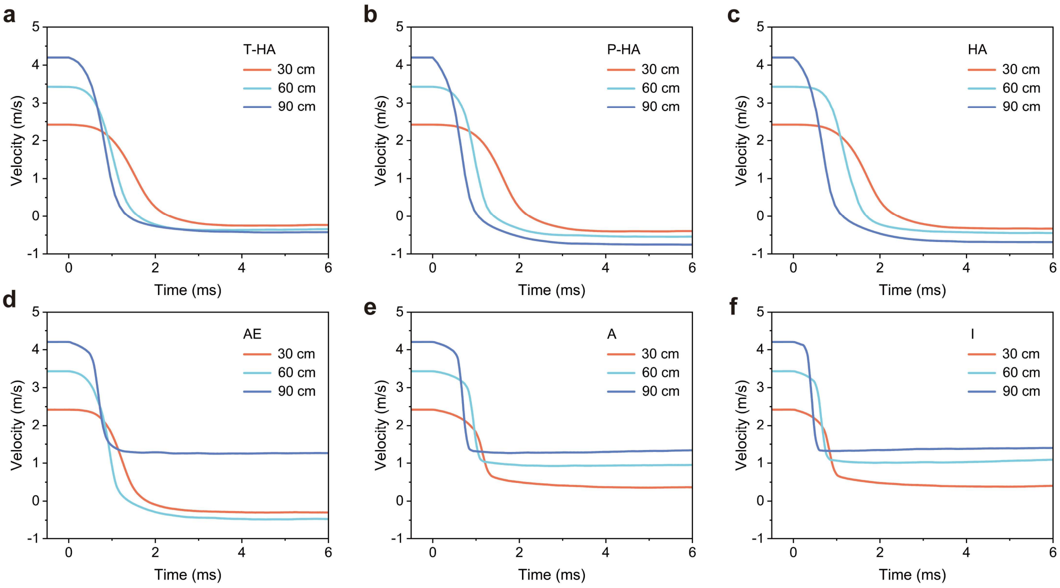

Fig. S6. Velocity-time curves of different samples during falling-ball impact at different heights.

Fig. S6 illustrates that a steel ball dropped from varying heights failed to penetrate HA hydrogels, P-HA, and T-HA composites, as evidenced by the negative final velocities recorded for the projectile. By comparison, when the impact height was fixed at 90 cm, the steel ball fully penetrated the I, A, and AE hydrogels, with positive final velocities verifying complete perforation. These observations are substantiated by SEM images of the front and back surfaces of impacted specimens (Fig. S7). For the I hydrogel, large through-holes were detected on both surfaces after impact, demonstrating a lack of effective impact resistance. The A hydrogel, despite being fabricated via directional freeze-casting, still suffered extensive structural degradation under impact loading. In contrast, the AE hydrogel exhibited drastically reduced hole formation on both surfaces, a result that validates the efficacy of combining directional freeze-casting with salting-out to improve the impact tolerance of hydrogels.

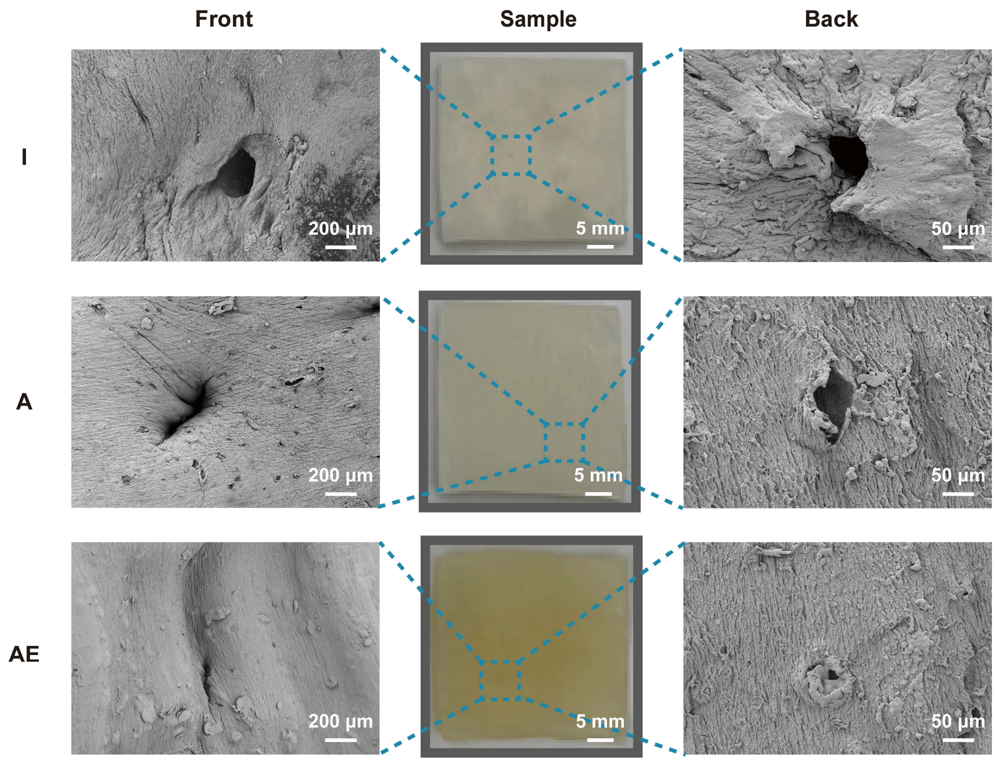

Fig. S7. Surface perforation morphologies of different hydrogel samples after falling-ball impact (I and A hydrogels at 30 cm height, AE hydrogel at 90 cm height).

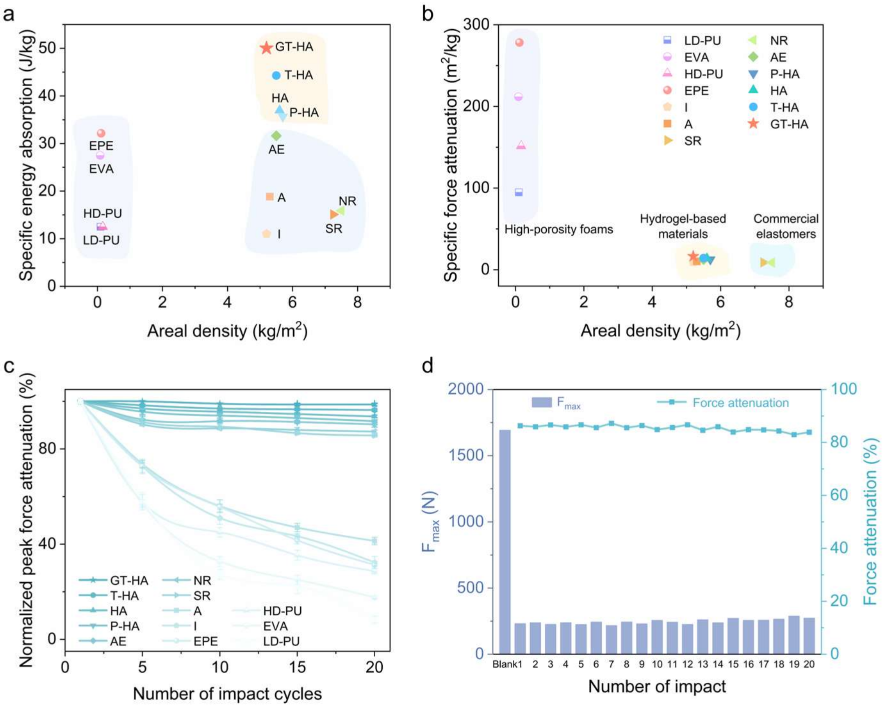

Fig. S8. SEA (a) and force-attenuation (b) of different samples normalized by areal density; Peak-force retention of different samples after 20 consecutive impacts (c); Force and attenuation rate of the composite under 20 cyclic impacts (d).

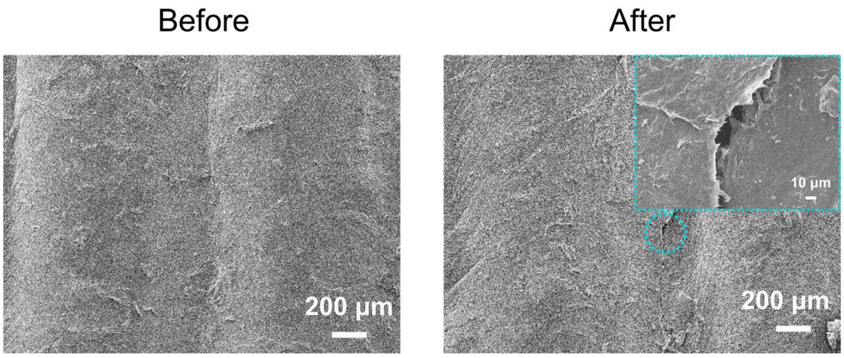

Fig. S9. SEM images of GT-HA composite before and after 20 cyclic impact tests.

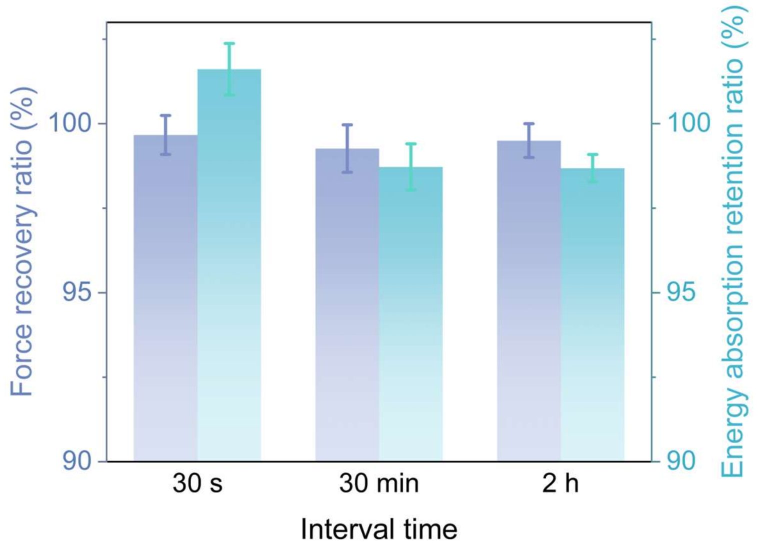

Fig. S10. Force recovery ratio, and energy absorption retention after different resting intervals.

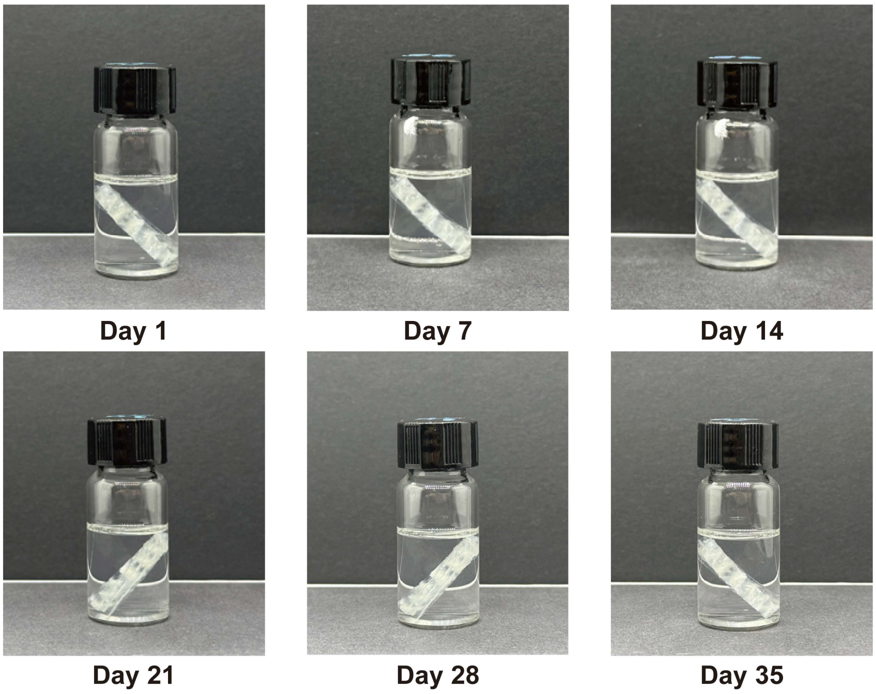

Fig. S11. Images of GT-HA composites immersed in water for 35 days.

To evaluate the long-term stability of GT-HA composites, the samples were immersed in deionized water for 35 days, with their morphological and structural states monitored throughout the period. As shown in Fig. S11, the composites exhibited no discernible swelling and retained a stable morphology over the entire 35-day period, and the soaking water remained clear and colorless. These observations confirm that the GT-HA composite material did not degrade during the experiment and exhibits excellent long-term stability.

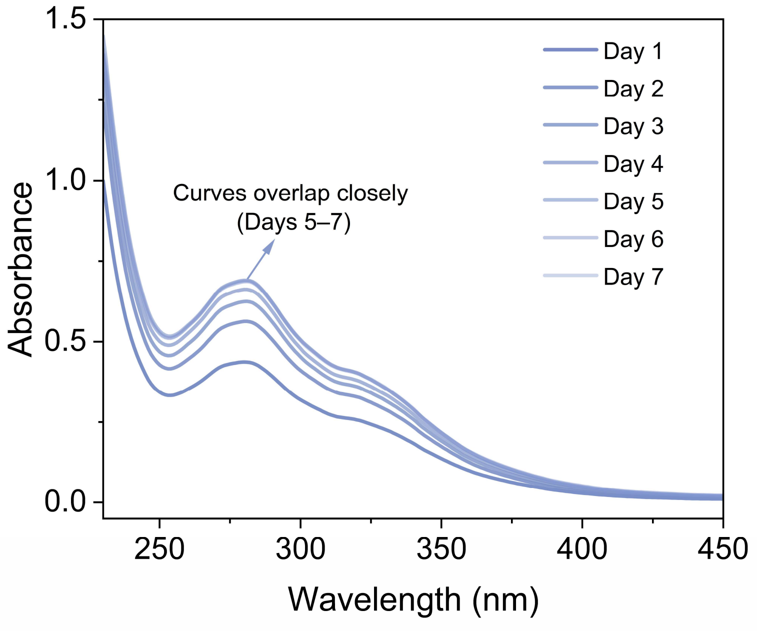

Fig. S12. UV-Vis spectra of leachate from GT-HA composite during 7 consecutive days of water immersion.

The initial water content of the GT-HA composite was 30.00%. Its initial mass was 0.84 g, with macroscopic dimensions of 13.50 × 10.00 × 4.50 mm^3. After 7 days of immersion, the sample reached swelling equilibrium. The final mass stabilized at 0.55 g, and the dimensions changed to 15.20 × 11.75 × 5.30 mm^3. The volumetric swelling ratio was only 55.86%, demonstrating excellent resistance to excessive swelling and reliable macroscopic structural stability. A water immersion test essentially characterizes the swelling and medium diffusion behavior of polymer networks. Water absorption of hydrogels and diffusion of free chain segments generally reach thermodynamic equilibrium within several days, so a 7-day immersion period was adopted. The aforementioned 35-day test was carried out to evaluate long-term aging resistance and cyclic impact performance under ambient conditions. The 7-day dynamic UV-Vis monitoring (Fig. S12) further verifies this conclusion: the spectra from Days 5 to 7 nearly overlapped, and the absorbance increment approached zero, indicating that

leached components had reached dynamic equilibrium. Therefore, the 7-day period is sufficient to confirm that the system has reached a stable chemical state. Notably, the sample mass decreased by approximately 34.50% after immersion, which corresponds well with the gradual rise in absorbance within the first three days of UV-Vis testing. This mass loss mainly results from the complete dissolution of sodium chloride trapped in the network during salting-out, along with the loss of a small amount of free uncrosslinked chains on the material surface. Since NaCl shows no characteristic UV absorption, the overall absorbance remains low. This rules out large-scale structural disintegration of the matrix network.

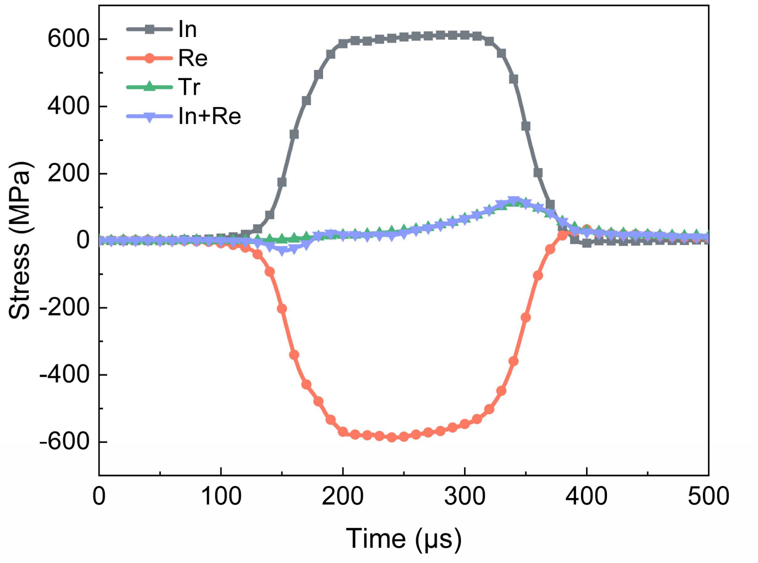

Fig. S13. Verification of dynamic stress equilibrium.

We selected the original waveform corresponding to a peak strength of 183.57 MPa and a strain rate of approximately 4000 s^{-1} and calculated the stress at the specimen's incident face (incident+reflected waves, In+Re) and transmitted face (transmitted wave, Tr). As shown in Fig. S13, during the entire loading plateau and around the peak region, the In+Re and Tr stress curves nearly coincide, confirming that the specimen maintains dynamic stress equilibrium throughout the test, ensuring the validity of the measurements.

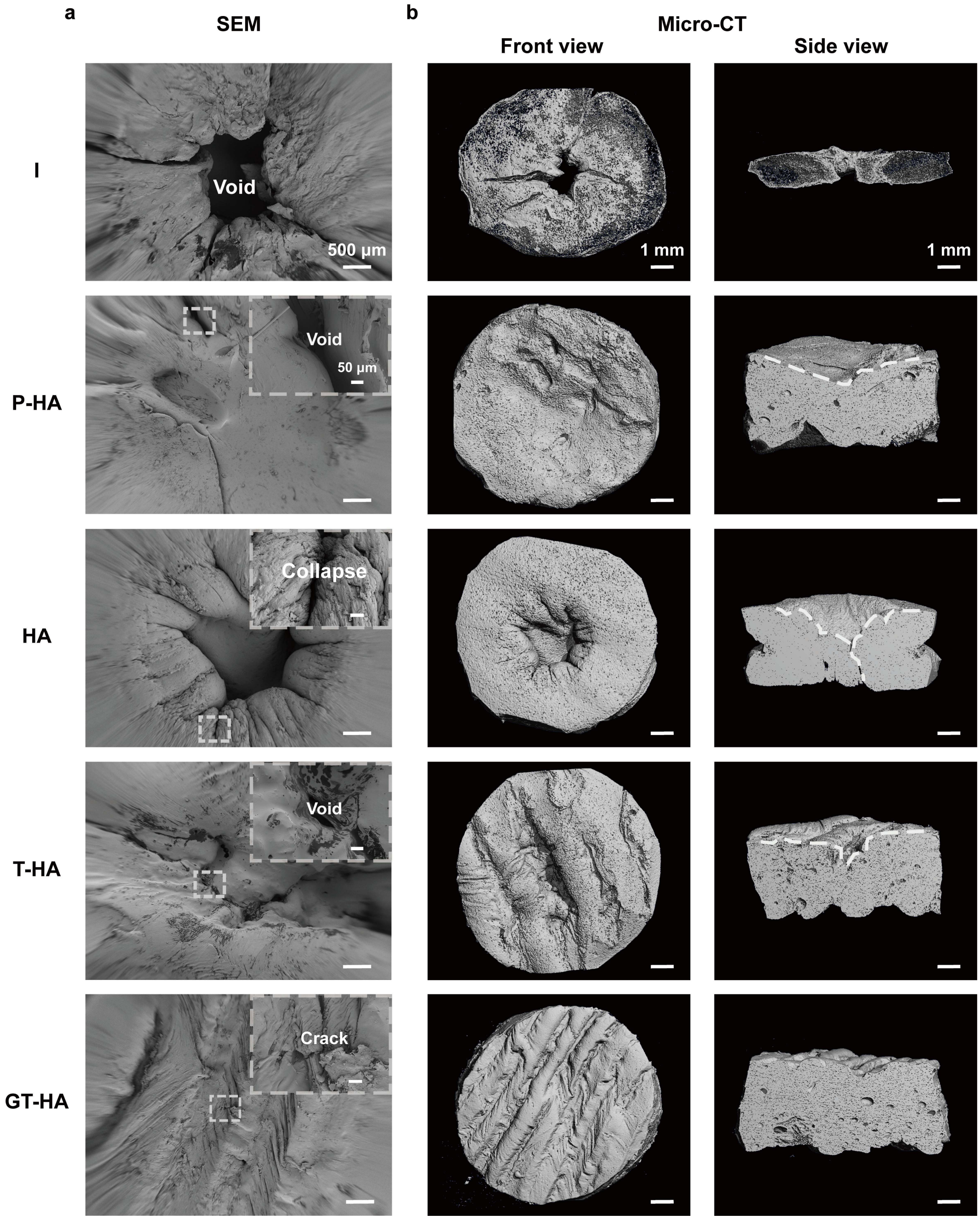

Fig. S14. SEM (a) and micro-CT images (b) of different samples after SHPB testing (strain rate: 2800 s^{-1}).

Fig. S14 presents SEM (a) and micro-CT images (b) of I, HA, P-HA, T-HA, and GT-HA samples after SHPB testing (strain rate: 2800 s^{-1}). The results show that the I hydrogel, due to its heterogeneous internal network structure, forms a large perforation directly upon impact, exhibiting the poorest mechanical performance. In the P-HA composite, the P framework induces stress concentration and disrupts the microstructure order, preventing effective regulation of the macro-microscale competition (MMC) mechanism. Its SEM images reveal distinct indentations and large-

scale pores (indicated by dashed boxes), which are further corroborated by micro-CT results. The HA hydrogel surface shows noticeable depression and cracking (dashed boxes), indicating that despite some energy-absorption capacity provided by its micro-scale dissipation mechanisms, the sample still undergoes severe damage. SEM images of the T-HA composite display relatively small pores (dashed boxes), but are accompanied by extended surface cracks. Corresponding micro-CT results confirm substantial surface damage. This is because the T framework, although improving crack deflection, does not fully integrate gradient stress guidance. In contrast, the GT-HA composite exhibits only shallow surface cracks (dashed boxes), with the GT framework remaining largely intact and showing minimal damage. This demonstrates that the specially designed GT framework, through coordinated regulation of the MMC mechanism, achieves an integrated macro- and micro-scale energy dissipation, thereby delivering optimal impact-resistant performance.

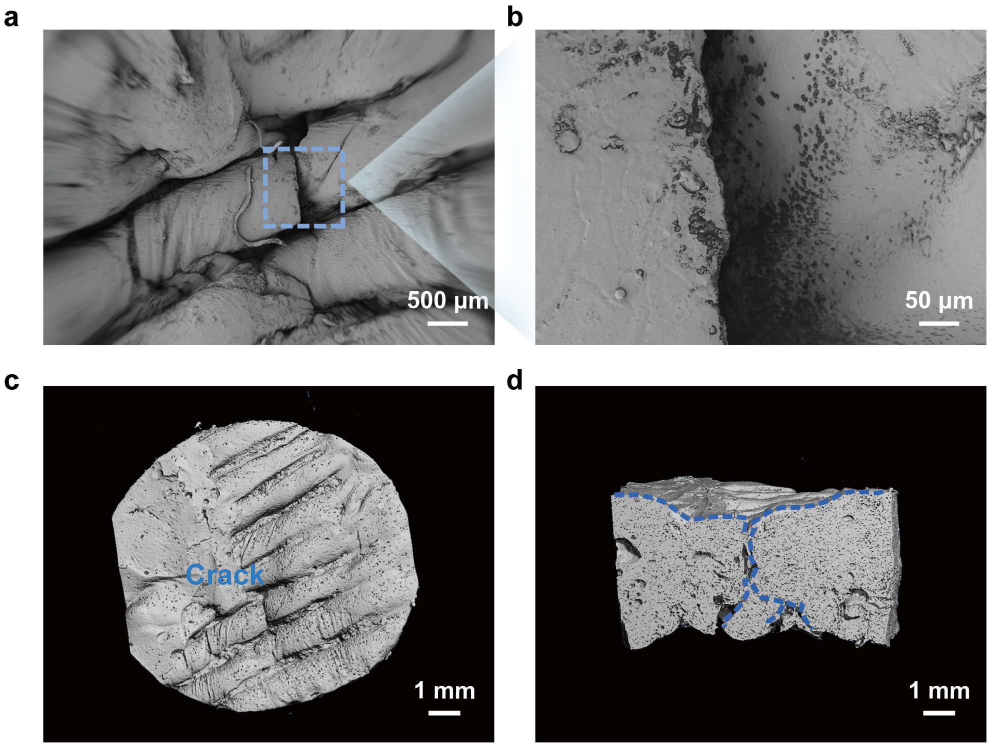

Fig. S15. SEM (a, b) and micro-CT images (c, d) of GT-HA composites after SHPB testing (strain rate: 3500 s^{-1}).

To verify the impact resistance of GT-HA composites under high-speed impact conditions, SHPB tests were performed at a strain rate of 3500 s^{-1}. Fig. S15 clearly presents the morphological characteristics and damage states of GT-HA composites after SHPB impact. From the SEM and micro-CT front views (Fig. S15a-c), obvious depressions and fractures can be observed in the surface GT frameworks; the micro-CT

side view (Fig. S15d) shows that the impact cracks have a relatively large depth, indicating that the samples suffered a certain degree of damage upon impact. Notably, the impact propagation path of the stress wave in the side view exhibits significant tortuosity, effectively reducing the penetration velocity of the impactor. This confirms that the rationally designed GT framework enables efficient modulation of the MMC mechanism: its gradient and spiral geometry coordinates macroscale stress guidance and crack deflection with micro/nanoscale nanometer fiber sliding, nanocrystalline formation, and dynamic molecular interactions, thereby collectively endowing the GT-HA composite with exceptional impact resistance.

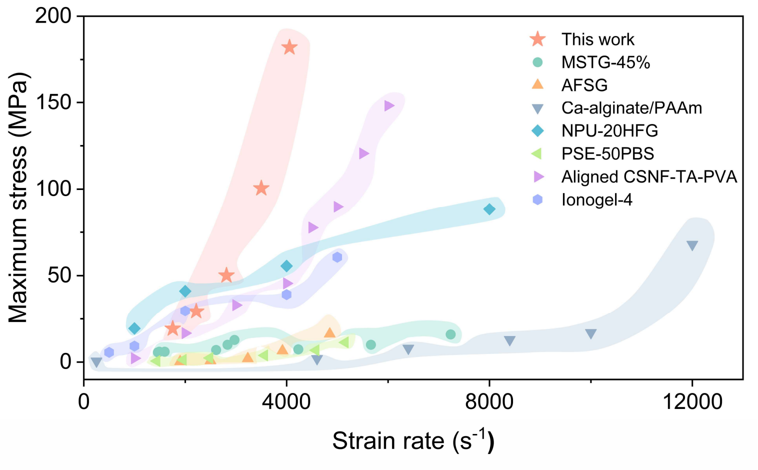

Fig. S16. Stress comparison between GT-HA composites and previously reported impact-resistant materials at different strain rates.

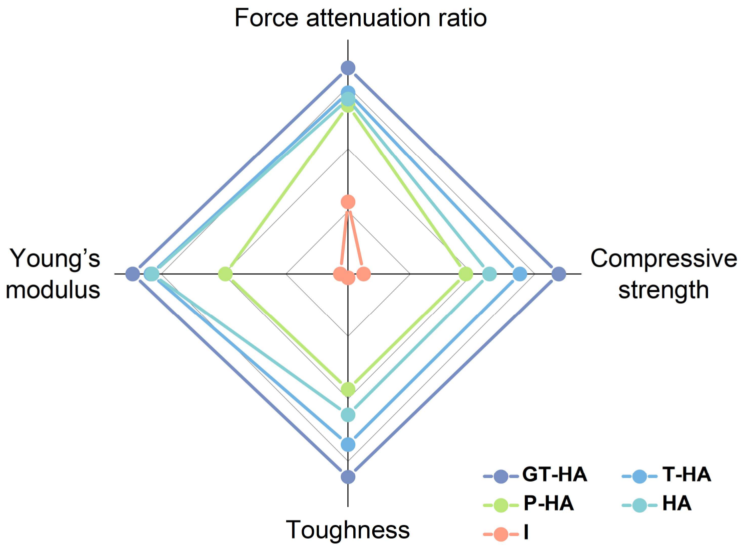

Fig. S17. A radar map comparing the mechanical properties of GT-HA, T-HA, P-HA composites, HA, and I hydrogel, including force attenuation ratio, compressive strength, toughness, and Young's modulus.

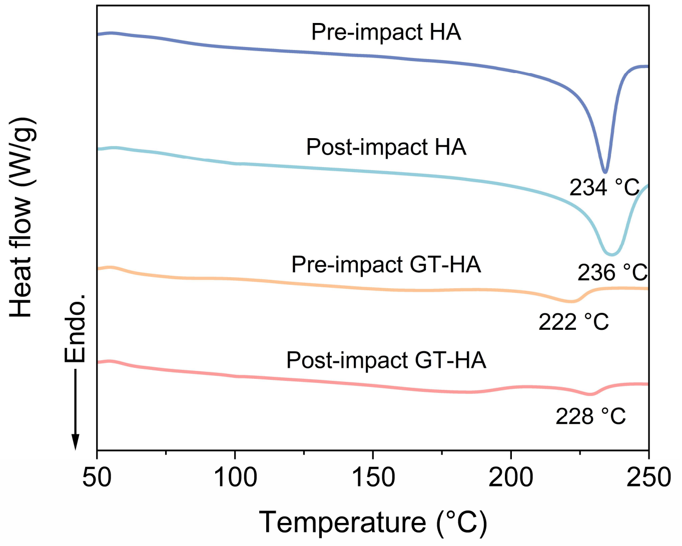

Fig. S18. DSC characterization of HA hydrogel and GT-HA composite before and after SHPB impact (strain rate: 2800 s^{-1})

We performed DSC tests on dried pure HA and GT-HA samples before and after SHPB impact at a strain rate of 2800 s^{-1}. The total area of melting peaks remained nearly unchanged for both groups, indicating no new crystals formed during the transient impact. Obvious shifts in melting temperature confirmed strain-induced network reconfiguration. The initially lower melting temperature of GT-HA was attributed to disrupted crystalline order caused by interfacial pinning between the framework and matrix. After impact, pure HA showed only a slight rise in melting temperature, while GT-HA exhibited a 6 °C right shift of the melting peak. This suggests polymer chain re-entanglement occurred under framework confinement and shear, leading to a denser microstructure and improved thermal stability. These results thermodynamically verify the macro-micro competition mechanism of the material.

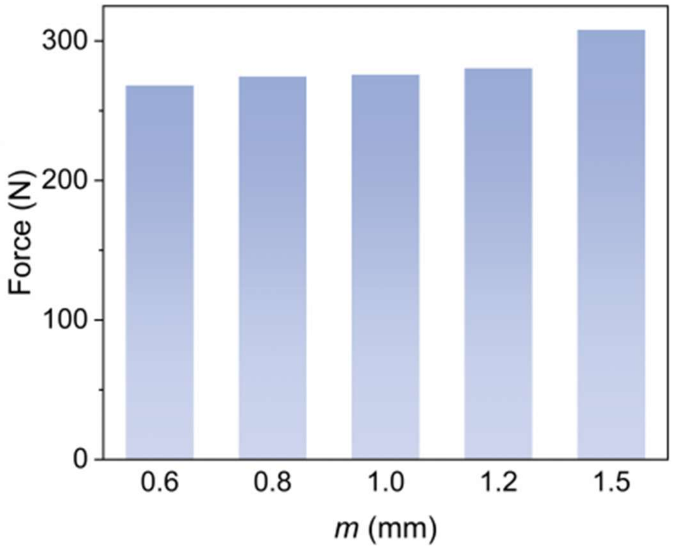

Fig. S19. Mesh sensitivity analysis of GT-HA composite FE model.

To exclude potential bias from spatial discretization and verify model independence, a mesh convergence study was conducted (Fig. S19). As the element size (*m*) was refined from 1.5 mm to 0.6 mm, system stress entered a stable convergence plateau at 0.8 mm, confirming mesh-independent results.

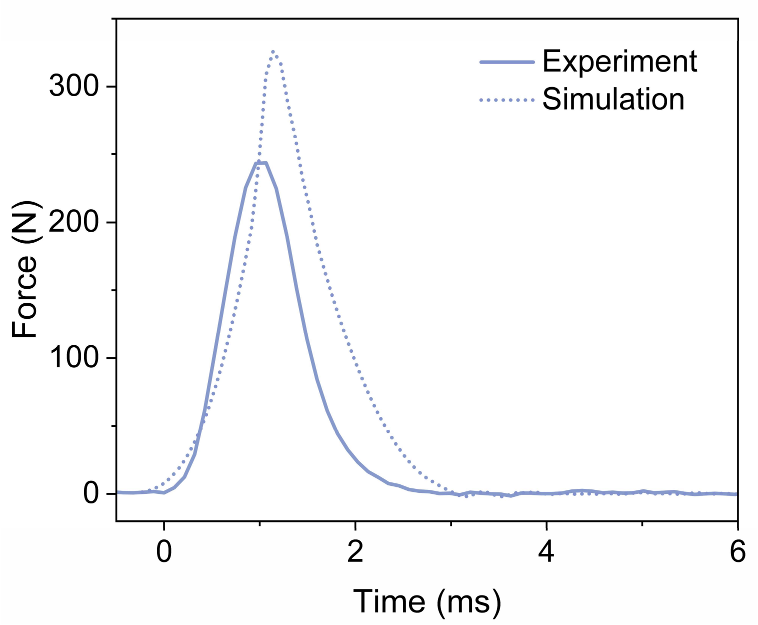

Fig. S20. Quantitative experimental validation of GT-HA composite at 4.2 m/s.

Quantitative validation via experimental force-time curves. To verify the robustness of the revised constitutive framework and contact algorithm, we first performed a quantitative comparison between numerical predictions and experimental measurements at a feasible impact velocity of 4.2 m/s (Fig. S20). Benefiting from the physically informed interfacial delamination behavior introduced in the revised model, the predicted peak impact force and buffering duration agree well with the experimental curves. This demonstrates the high reliability of the present constitutive framework and parameter calibration strategy, which validates its applicability for subsequent numerical analysis of ultra-high-speed impact at 50 m/s.

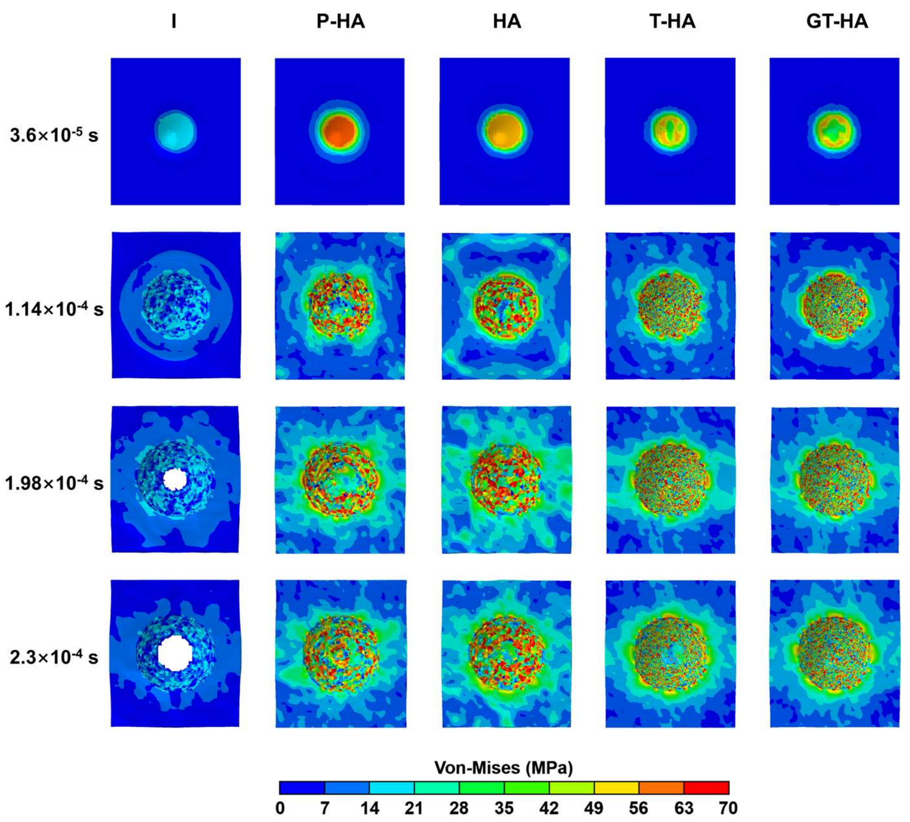

Fig. S21. Stress distribution of I hydrogels, P-HA composites, HA hydrogels, T-HA composites, and GT-HA composites during the impact process.

The energy dissipation mechanism of I, P-HA, HA, T-HA, and GT-HA samples during impact was investigated through finite element (FE) simulation of the falling-ball impact process under identical conditions. Fig. S21 clearly illustrates the damage evolution over time for the five samples, revealing consistent simulation results with experimental impact data. I hydrogels underwent complete perforation after an impact of 1.98×10^{-4} s and suffered catastrophic damage, indicating the poorest protective effect. The P-HA composite underwent perforation due to the failure of the MMC mechanism. Although HA hydrogels remained non-penetrated under high-velocity impact, they displayed pronounced stress concentration. The T-HA achieves crack deflection through the embedded T framework but fails to optimize stress distribution due to insufficient framework complexity. In contrast, the GT-HA composite, benefiting from the deliberate design of its biomimetic frameworks, effectively exploited the MMC mechanism to enhance impact resistance. Further analysis of Fig. S21 indicates that GT-HA composites achieved uniform load transfer and superior protective performance, demonstrating that the structural design of biomimetic frameworks plays a pivotal role in governing the impact resistance of composites. These results demonstrate that coupling macroscopic topological design with microscopic energy

dissipation mechanisms, regulated via the MMC mechanism, can further optimize stress distribution, enhance dynamic toughness, and ultimately achieve multi-level energy absorption.

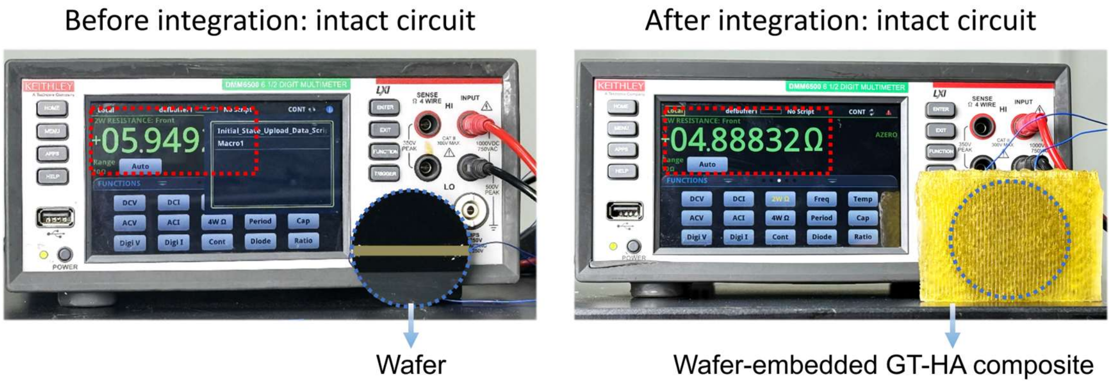

Fig. S22. Electrical continuity of electrodes before and after composite integration

To examine whether the entire fabrication process (directional freezing, annealing and salting-out) would damage pre-fabricated microelectronic structures, gold (Au) electrodes were pre-deposited on the silicon wafer. The electrical continuity of electrodes was characterized using a source meter before and after composite integration (Fig. S22). The results reveal that the electrodes retained complete circuit continuity and stable conduction. This demonstrates that the fabrication process does not damage or degrade pre-deposited metal electrodes, and shows excellent compatibility with wafer-scale microelectronic integration technology.

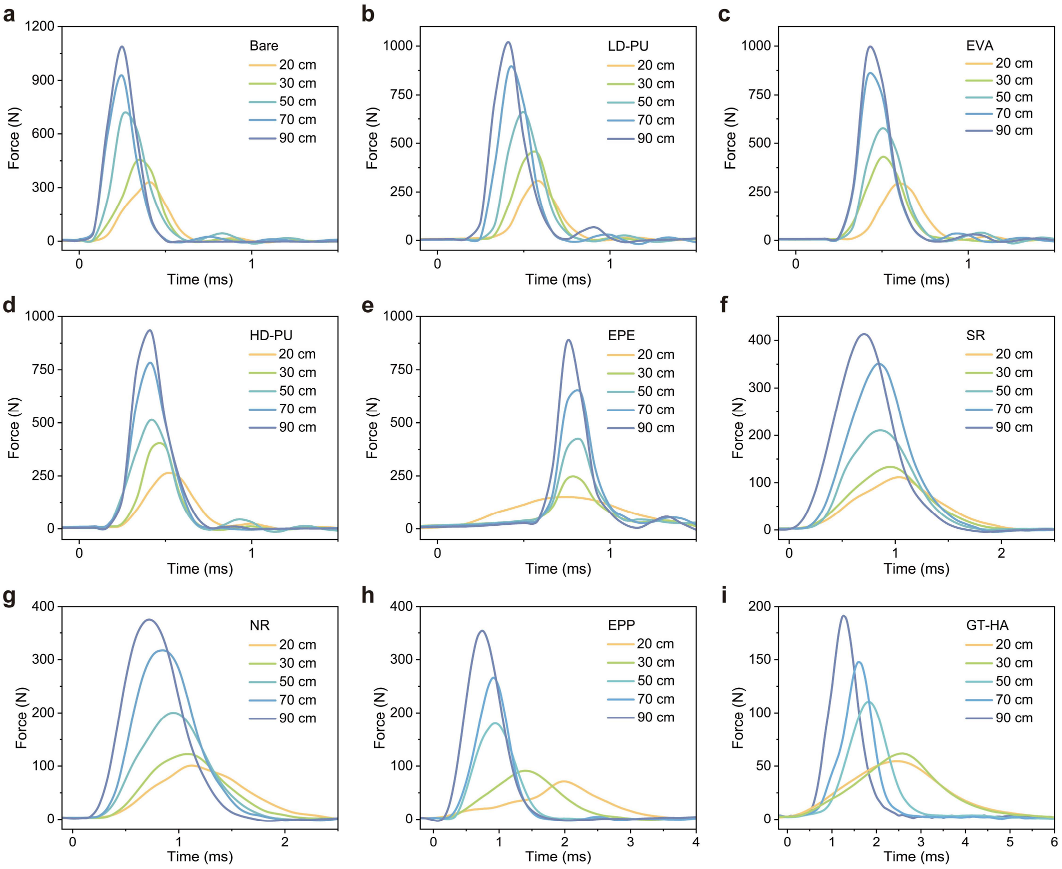

Fig. S23. Force-time curves of different samples for glass protection at heights ranging from 20 to 90 cm. a-i, These impact actions were conducted on (a) Bare, (b) LD-PU, (c) EVA, (d) HD-PU, (e) EPE, (f) SR, (g) NR, (h) EPP, and (i) GT-HA, respectively. All specimens had a dimension of 35×35×5 mm^3.

Fig. S23 shows that in the falling-ball impact test on protective glass, the impact force endured by different samples increases with the rise in the falling height of the steel ball. Meanwhile, the peak impact force data at heights from 20 to 90 cm reveal a successive decreasing trend across all tested materials: Bare, LD-PU, EVA, HD-PU, EPE, SR, NR, EPP, and GT-HA composites. Among these, the impact force attenuation ratio for GT-HA composites reaches 83%, significantly higher than the 68% of EPP, the best-performing control group. This result conclusively demonstrates the excellent impact resistance of GT-HA composites.

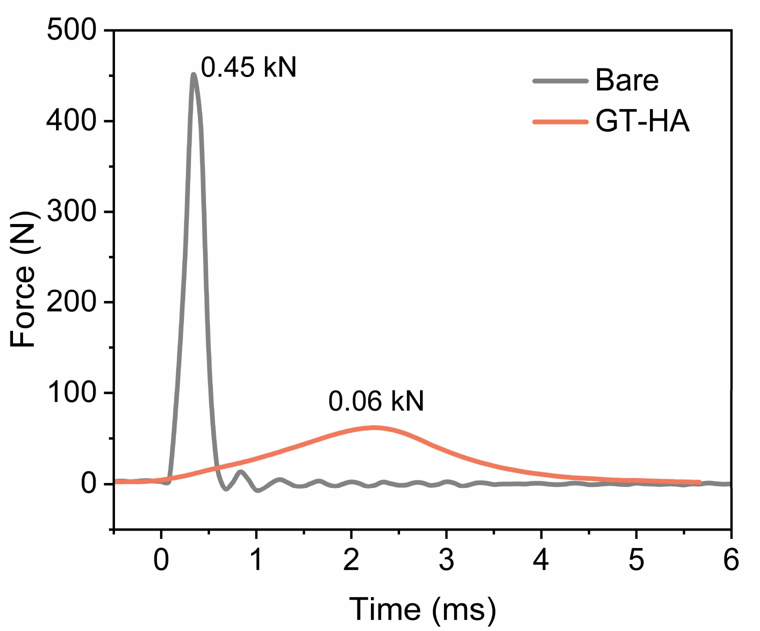

Fig. S24. Peak force curves of blank glass and GT-HA composite under an impact height of 30 cm.

Supplementary Table

Table S1. Peak fitting model for Fourier transform infrared spectroscopy (FTIR)

Sample	Fitting range (cm^{-1})	Baseline type	Peak assignment	Corrected R^2
I				0.9996
HA	3000-3700	Linear baseline	Hydrogen-bonded OH: 3170 cm^{-1} (strong), 3290 cm^{-1} (medium), 3400 cm^{-1} (weak); Free OH: 3490 cm^{-1}	0.9996
GT-HA				0.9986

Table S2. Comparison of stress and toughness between GT-HA composites and previously reported impact-resistant materials at different strain rates.

Samples	Maximum stress (MPa)	Energy absorption capacity (MJ/m^3)	Corresponding strain rate (s^{-1})	References
GT-HA composites	183.57	47.24	4056	This work
AFSG	16.24	1.96	4851	[57]
NPU-20HFG	88.3	13.45	8000	[52]
MSTG-45%	16	10.37	7236	[58]
Ca-alginate/PAAm	68	12.38	12000	[44]
Aligned CSNF-TA-PVA	148.2	36.08	6000	[18]
PSE-50PBS	11.40	3.40	5153	[53]
Ionogel-4	60.6	27.80	5000	[55]

Table S3. ALLIE values for different samples.

Sample	ALLIE (J)
Pure hydrogel	46.50
P framework	4.92
T framework	5.13
GT framework	5.31
P-HA composite	48.53
T-HA composite	56.51
GT-HA composite	58.29

Table S4. ALLIE and λ of GT-HA composite under different mesh sizes.

Mesh Size (mm)	$E_{composite}$ (J)	$E_{framework}$ (J)	$E_{hydrogel}$ (J)	λ
1.2	55.98	5.24	45.61	1.10
0.8	58.29	5.31	46.50	1.13
0.6	59.83	5.28	47.3	1.14

Table S5. ALLIE and λ of GT-HA composite under different interfacial friction coefficients.

Friction coefficient	$E_{composite}$ (J)	$E_{framework}$ (J)	$E_{hydrogel}$ (J)	λ
0.1	57.46	5.31	46.50	1.11
0.2	58.29	5.31	46.50	1.13
0.4	59.37	5.31	46.50	1.15

Supplementary Movies

Movie S1: SHPB tests of various samples at a strain rate of 2.8×10^3 s^{-1}

This movie records the SHPB test process of I hydrogels, HA hydrogels, and GT-HA composites at a strain rate of 2800 s^{-1}. Among them, I hydrogels were directly squeezed out of the bars under the first stress wave; HA hydrogels generated a strain of approximately 0.64 under the first stress wave, entering the unloading stage once the incident bar ceased. Subsequently, the reflected wave propagated in the reverse direction, and the second stress wave further compressed the samples, which ultimately led to most of them being squeezed out of the bars; in contrast, GT-HA composites produced a strain of only approximately 0.5 under the first stress wave, and after being further compressed by the second stress wave, part of the samples was squeezed out of the bars.

Movie S2: SHPB tests were conducted on HA hydrogels and GT-HA composites embedded with chips and flexible printed circuits (FPCs), respectively, at a strain rate of 2×10^3 s^{-1}.

This movie demonstrates the impact protection effects of HA hydrogels and GT-HA composites on chips and FPCs at a strain rate of 2000 s^{-1}. After two stress wave compressions, HA hydrogels with embedded chips exhibited partial extrusion from the bars and significant compressive damage, whereas GT-HA composites showed no such obvious damage. When FPCs were embedded, the light bulb in the HA hydrogel group changed from an illuminated state to an extinguished state, presumably due to impact-induced damage to the FPC solder joints; in contrast, the light bulb in the GT-HA composite group remained illuminated throughout, indicating that its FPC was undamaged. These results confirm that GT-HA composites possess superior impact protection capabilities.

Movie S3: Comparison of glass impact protection of GT-HA composites with traditional buffer materials (falling height, *H* = 90 cm)

This movie demonstrates the protective effect of different samples on glass with a thickness of 1 mm at a falling height of 90 cm. Among them, low-density polyurethane foam (LD-PU), ethylene-vinyl acetate copolymer foam (EVA), high-density polyurethane foam (HD-PU), expandable polyethylene foam (EPE), natural rubber (NR), silicone rubber (SR), and expanded polypropylene (EPP) all failed to prevent glass breakage, indicating that such traditional buffer materials are unable to effectively dissipate the high energy generated by steel ball impact. In contrast, the GT-HA

composite can effectively resist impact and inhibit glass breakage, exhibiting significantly superior impact protection performance compared to traditional buffer materials.

Movie S4: Protection of Glass Bottles, Ceramic Crucibles, and Quail Eggs from Breakage by GT-HA Composite When Dropped from 1.5 m

This movie demonstrates the impact protection performance of the GT-HA composite for fragile items. A comparative experiment was conducted by dropping glass bottles, ceramic crucibles, and quail eggs from a height of 1.5 m. Items dropped onto the floor were damaged, whereas those dropped onto the surface of the GT-HA composite remained intact. This result confirms that the GT-HA composite has significant effectiveness in the impact protection of fragile items.
