# Supplementary figures and images for: Supra-biomimetic Impact-Resistant Composites via Harnessing Macro–Microscale Competition
Source: Research (Wash D C). 2026 Jul 10;9:1358. doi: 10.34133/research.1358 (PMC13351122; doi:10.34133/research.1358)

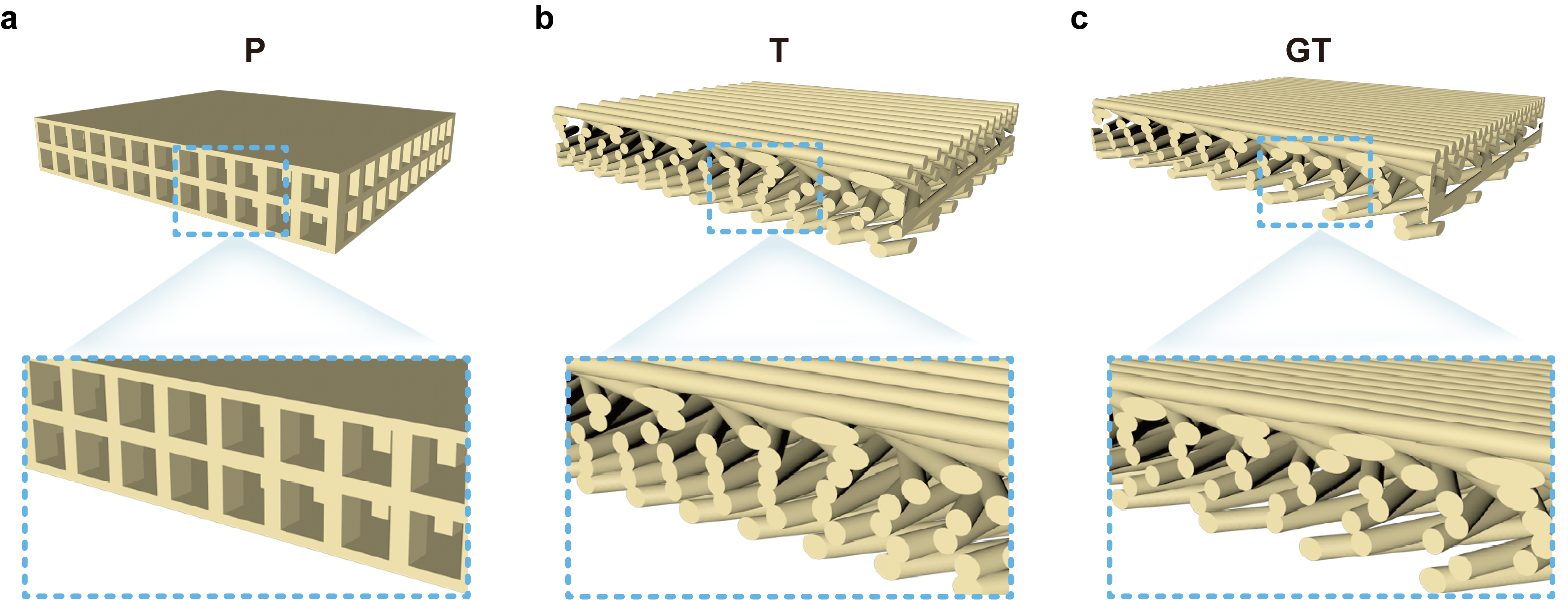

Supplement: Supplementary 1 — Figs. S1 to S24 Tables S1 to S5 Movies S1 to S4 [file research.1358.f1.zip › Fig. S1.tif]

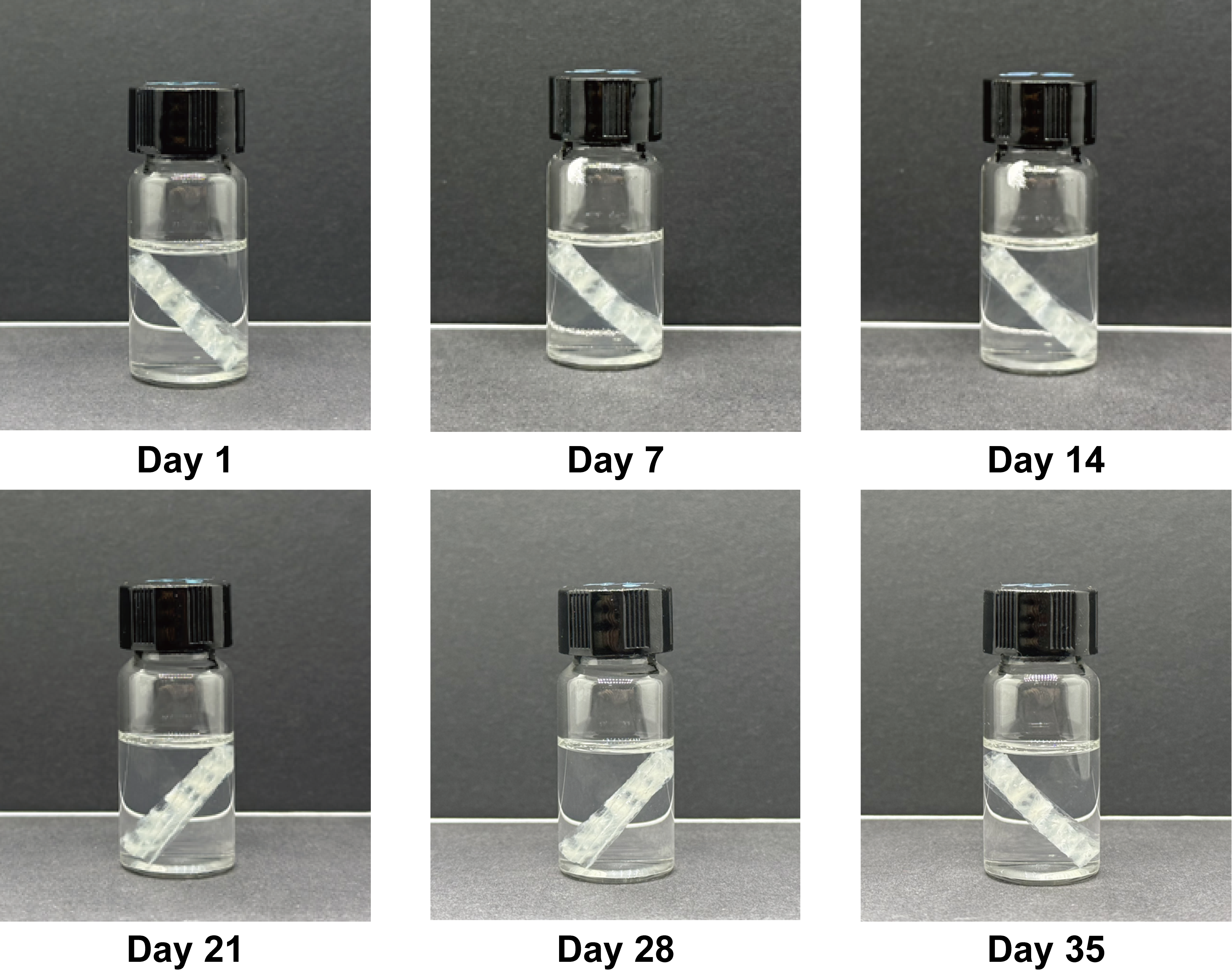

Supplement: Supplementary 1 — Figs. S1 to S24 Tables S1 to S5 Movies S1 to S4 [file research.1358.f1.zip › Fig. S11.tif]

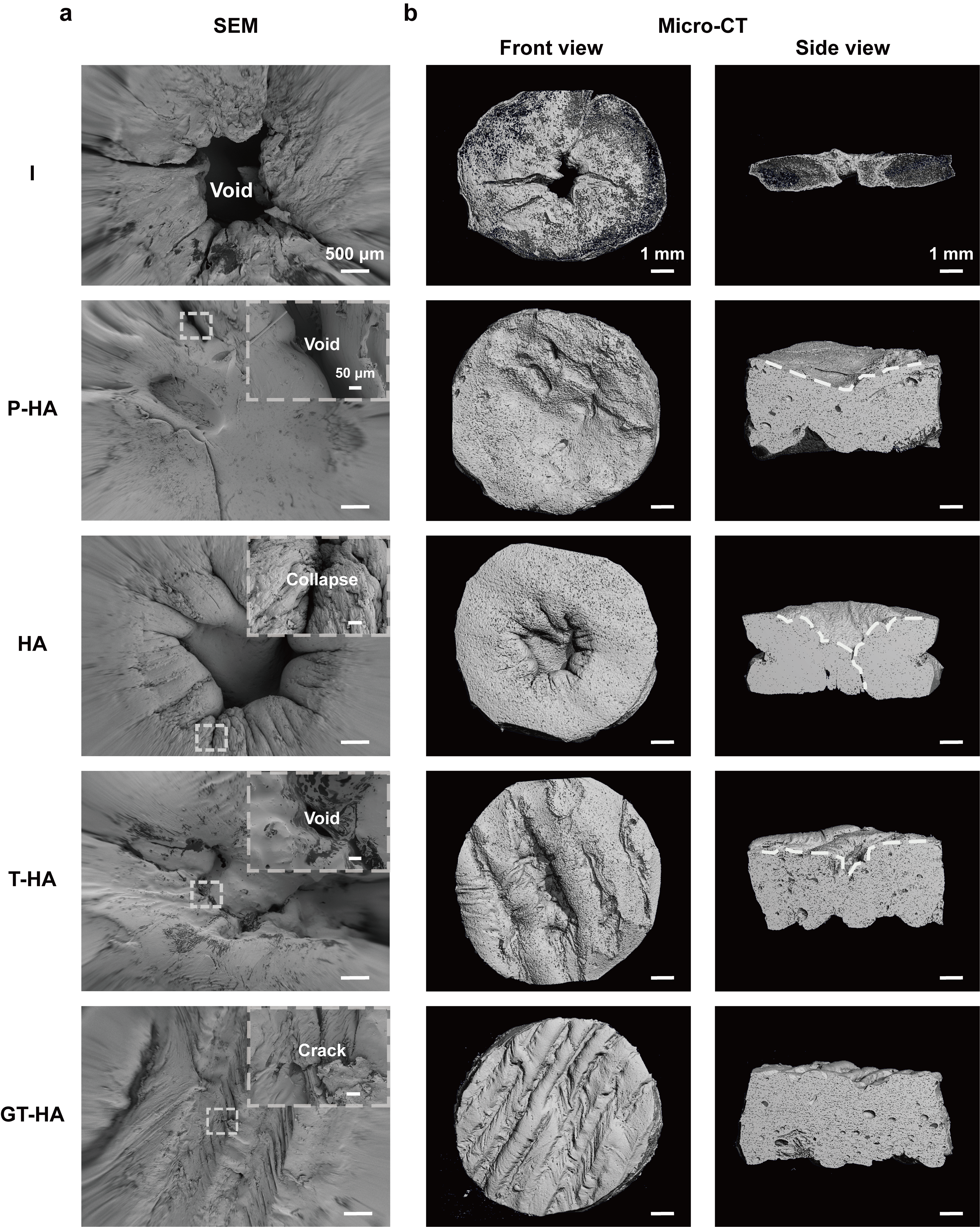

Supplement: Supplementary 1 — Figs. S1 to S24 Tables S1 to S5 Movies S1 to S4 [file research.1358.f1.zip › Fig. S14.tif]

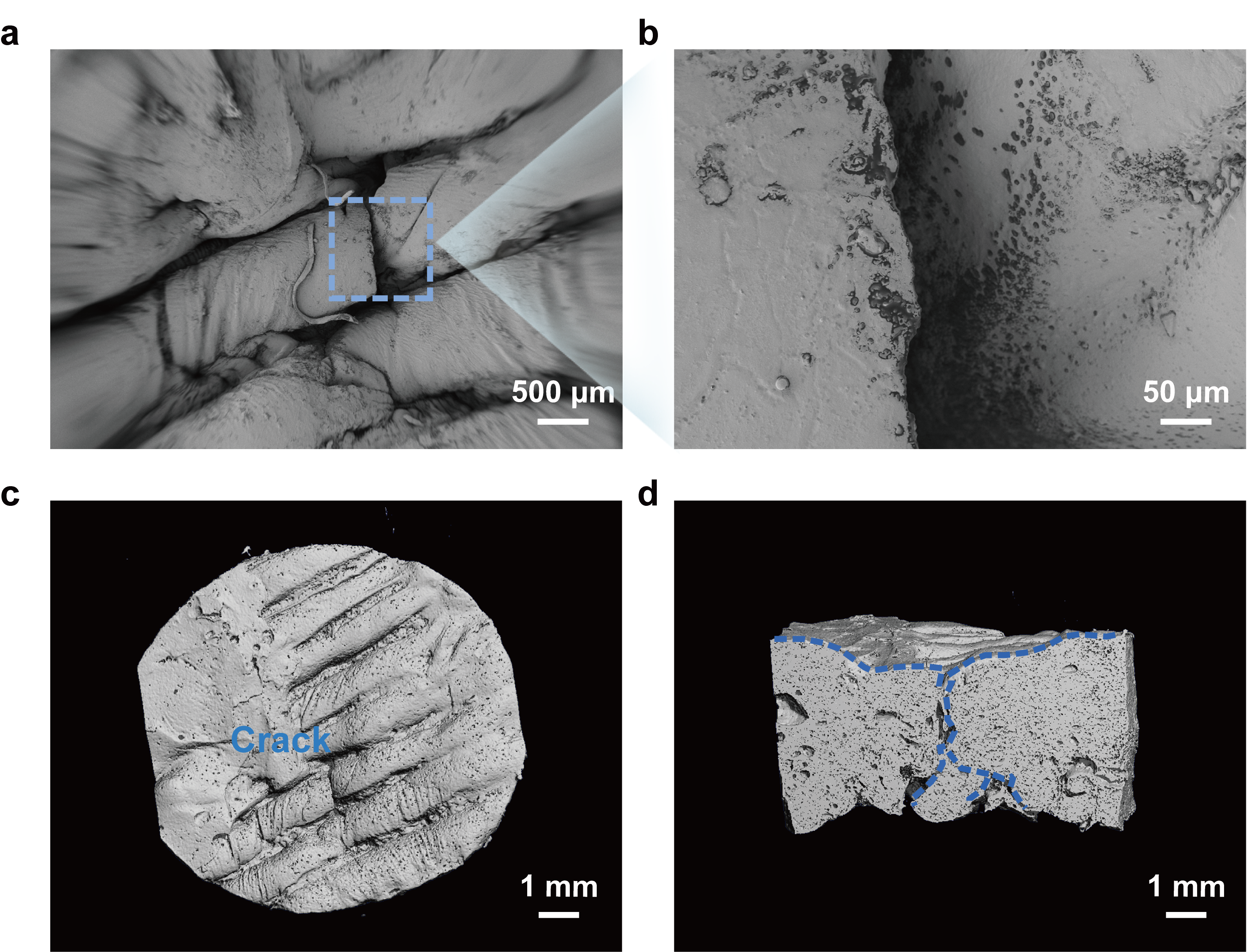

Supplement: Supplementary 1 — Figs. S1 to S24 Tables S1 to S5 Movies S1 to S4 [file research.1358.f1.zip › Fig. S15.tif]

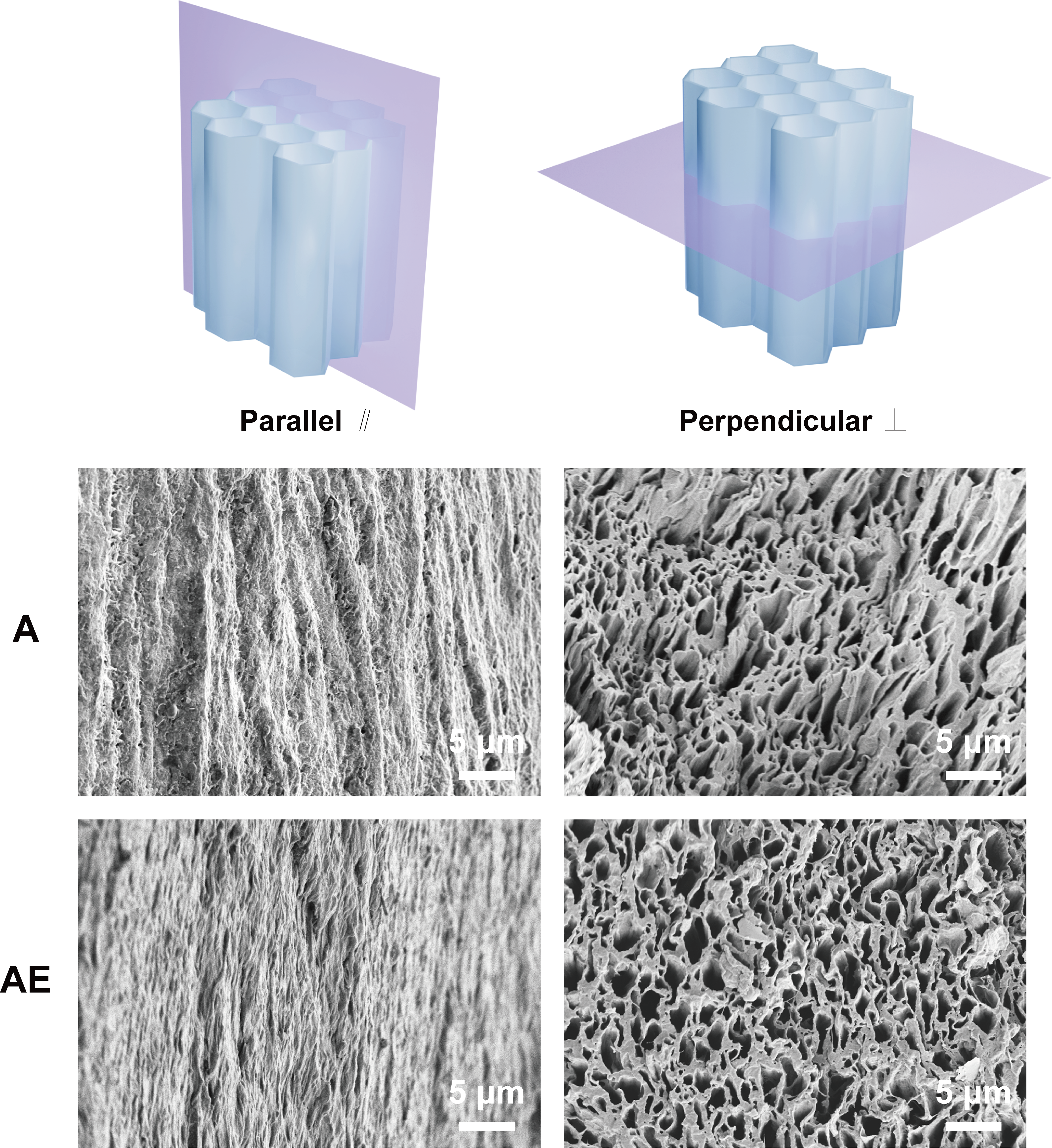

Supplement: Supplementary 1 — Figs. S1 to S24 Tables S1 to S5 Movies S1 to S4 [file research.1358.f1.zip › Fig. S2.tif]

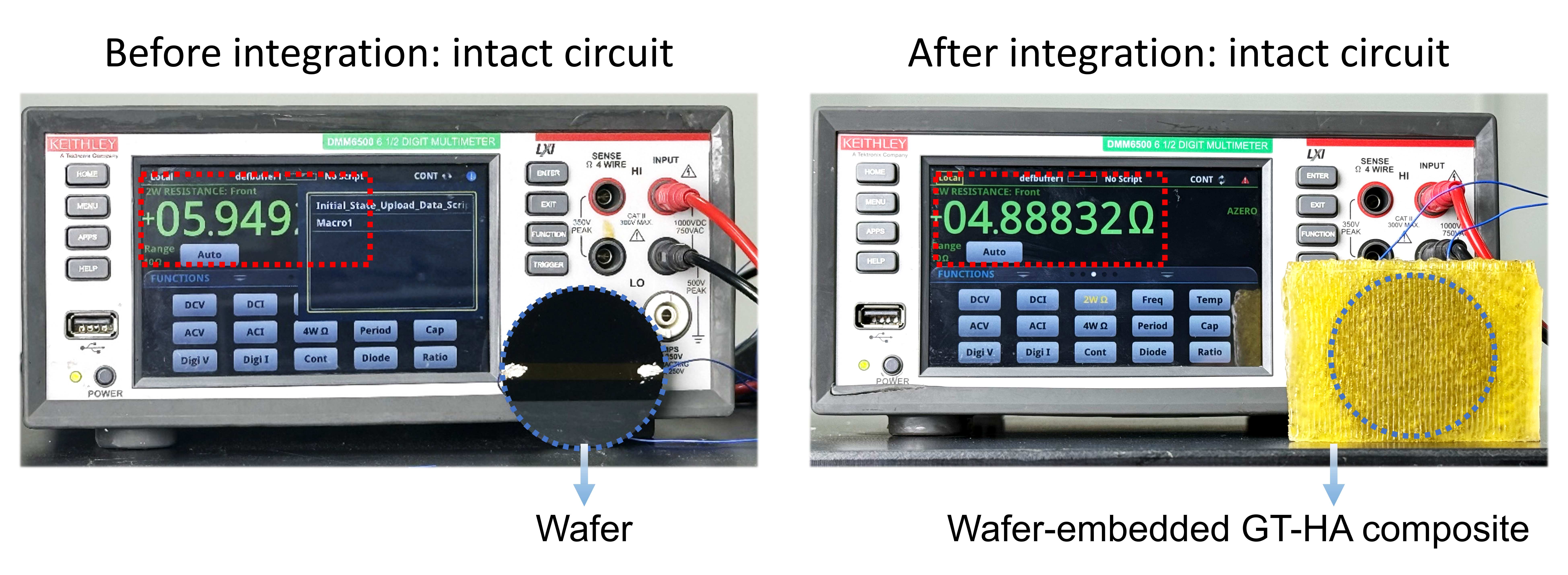

Supplement: Supplementary 1 — Figs. S1 to S24 Tables S1 to S5 Movies S1 to S4 [file research.1358.f1.zip › Fig. S22.tif]

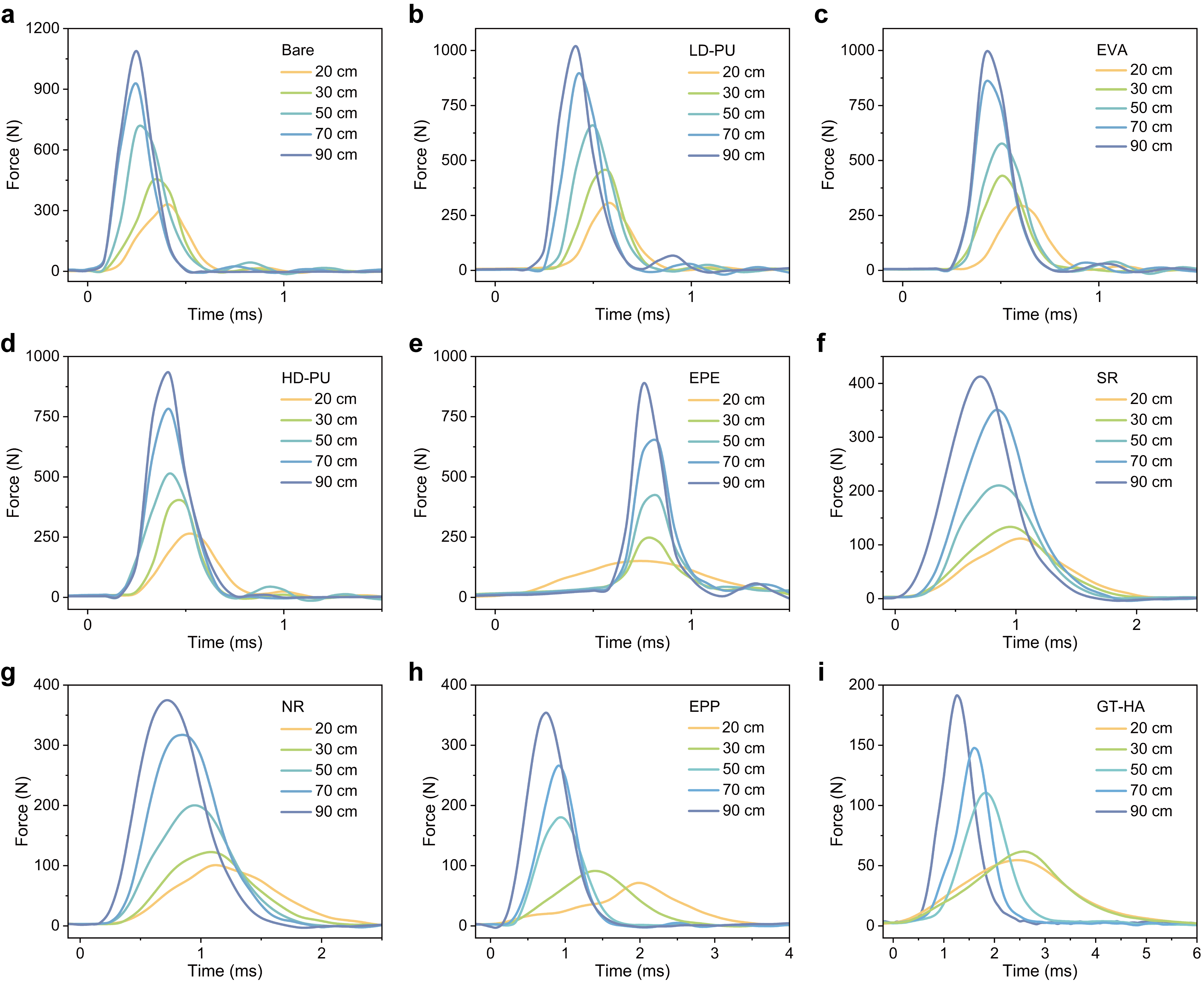

Supplement: Supplementary 1 — Figs. S1 to S24 Tables S1 to S5 Movies S1 to S4 [file research.1358.f1.zip › Fig. S23.tif]

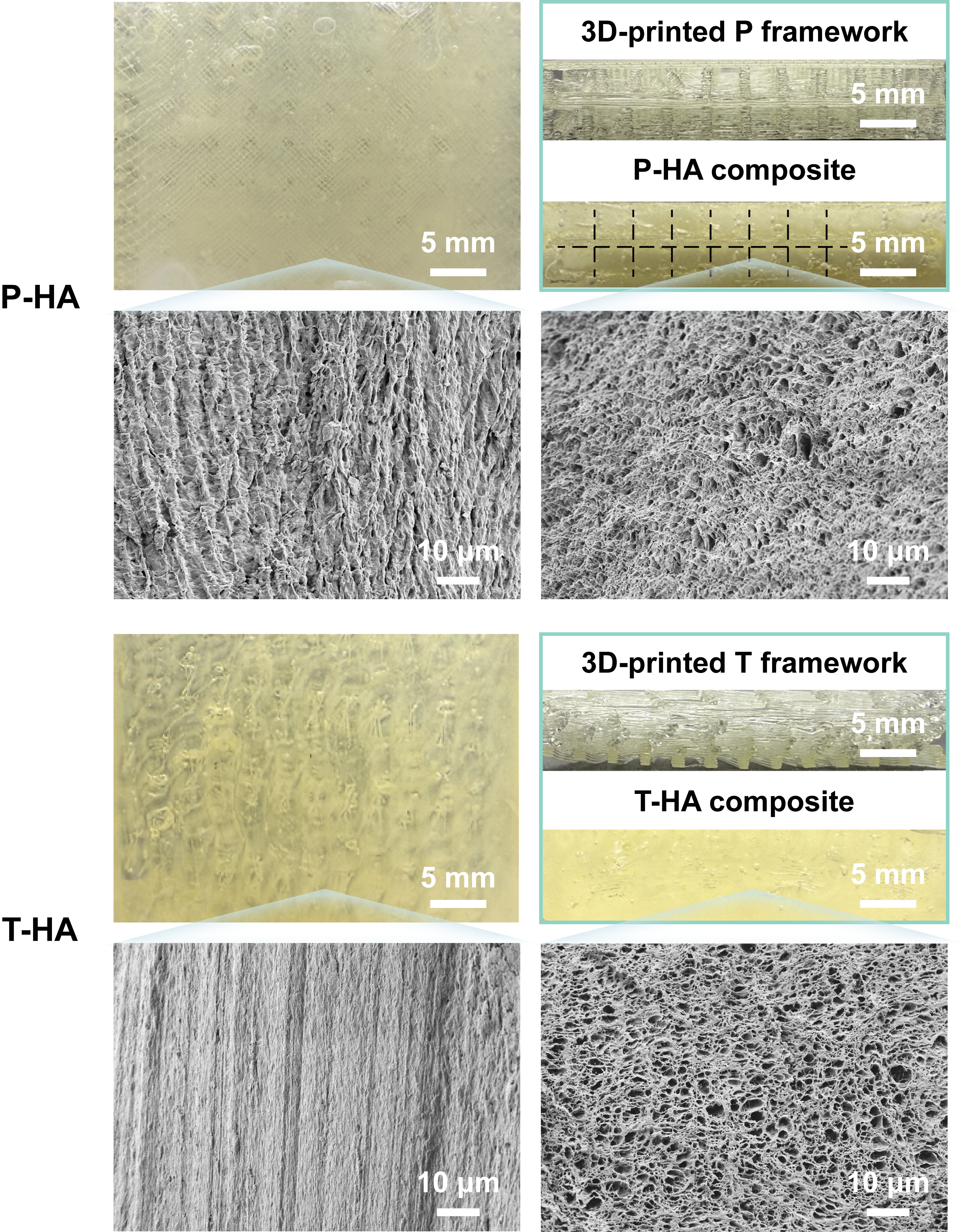

Supplement: Supplementary 1 — Figs. S1 to S24 Tables S1 to S5 Movies S1 to S4 [file research.1358.f1.zip › Fig. S3.tif]

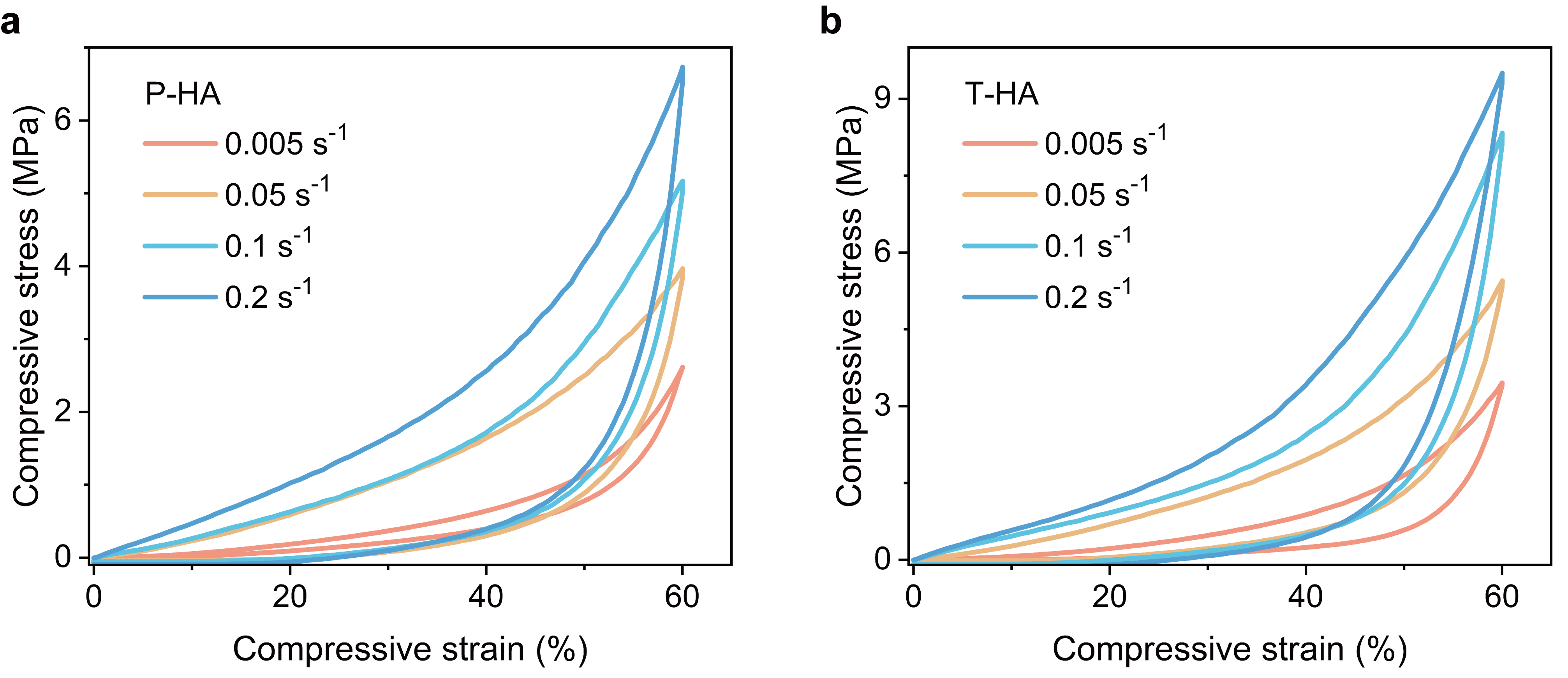

Supplement: Supplementary 1 — Figs. S1 to S24 Tables S1 to S5 Movies S1 to S4 [file research.1358.f1.zip › Fig. S4.tif]

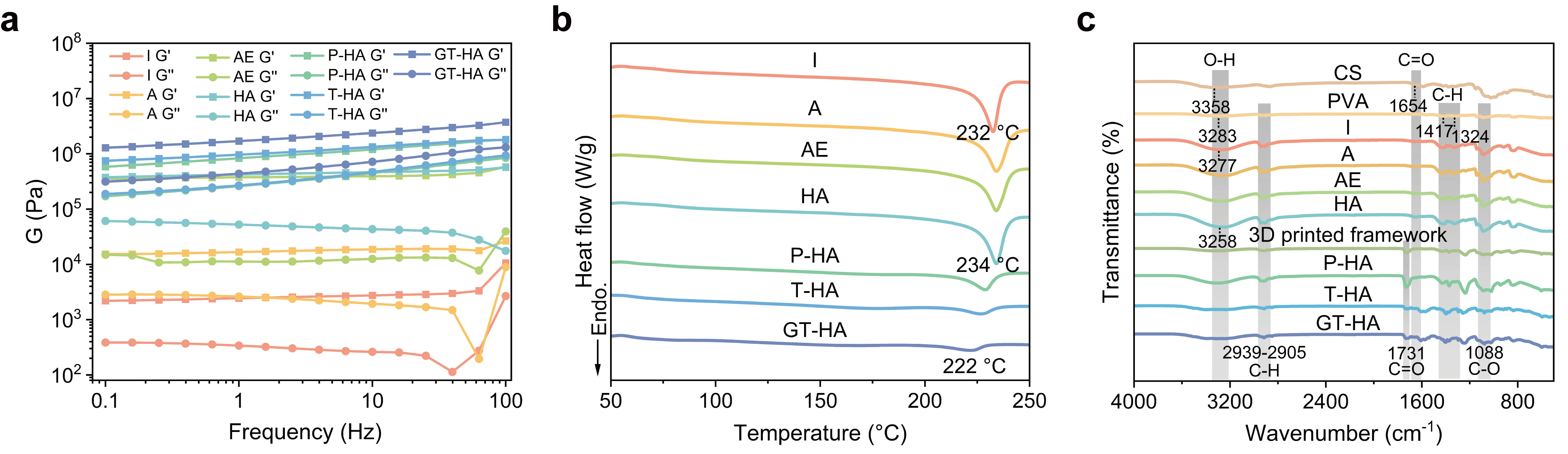

Supplement: Supplementary 1 — Figs. S1 to S24 Tables S1 to S5 Movies S1 to S4 [file research.1358.f1.zip › Fig. S5.tif]

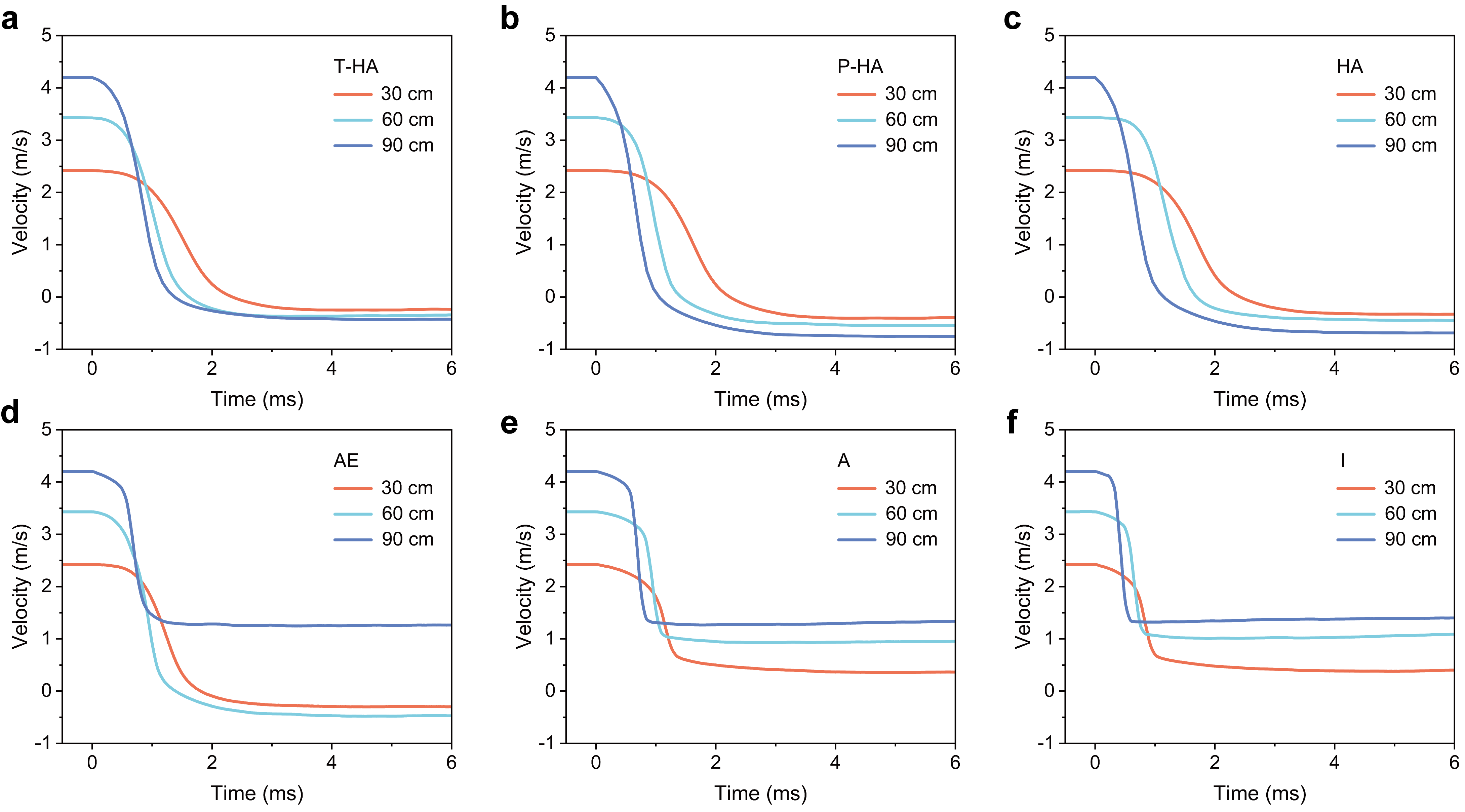

Supplement: Supplementary 1 — Figs. S1 to S24 Tables S1 to S5 Movies S1 to S4 [file research.1358.f1.zip › Fig. S6.tif]

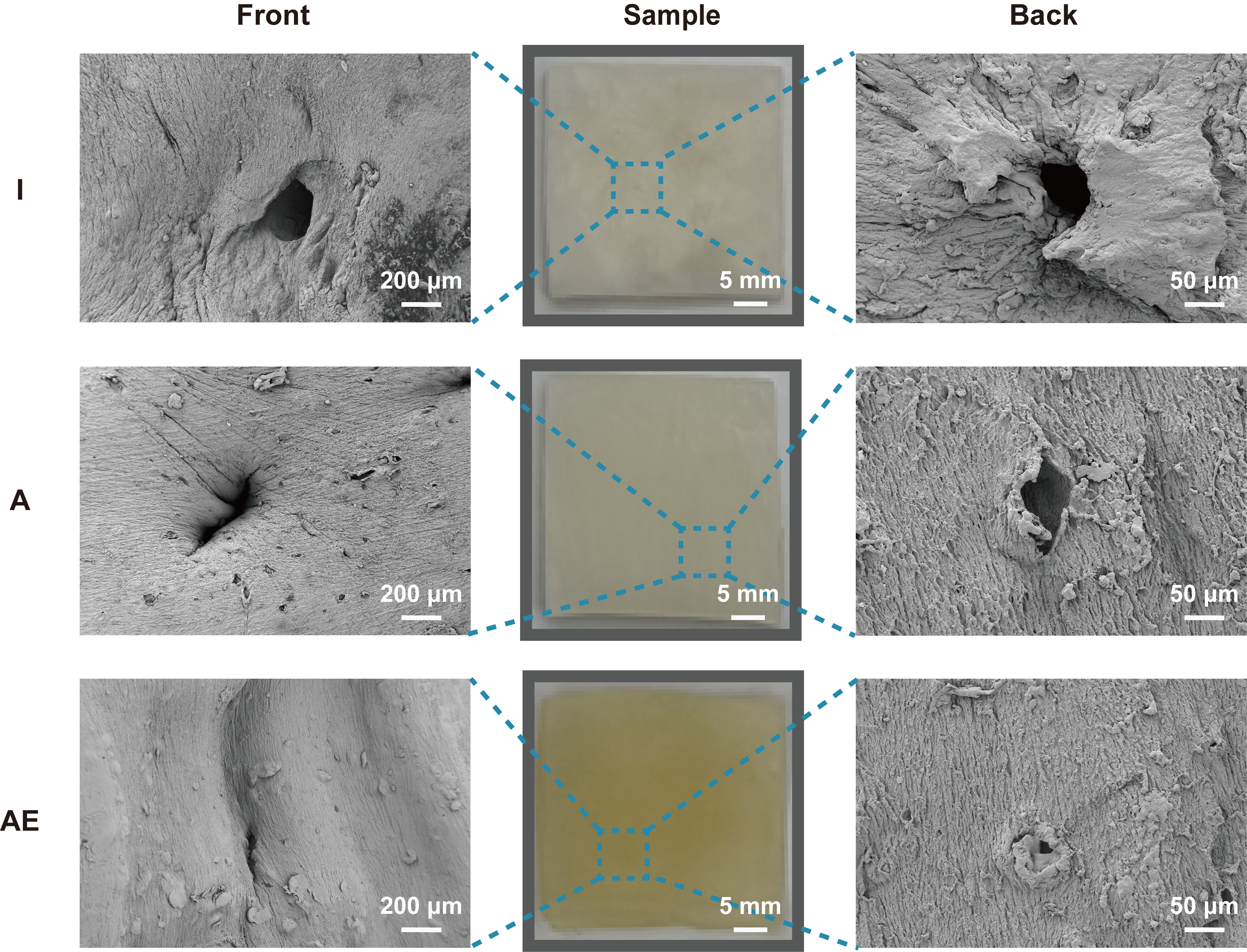

Supplement: Supplementary 1 — Figs. S1 to S24 Tables S1 to S5 Movies S1 to S4 [file research.1358.f1.zip › Fig. S7.tif]

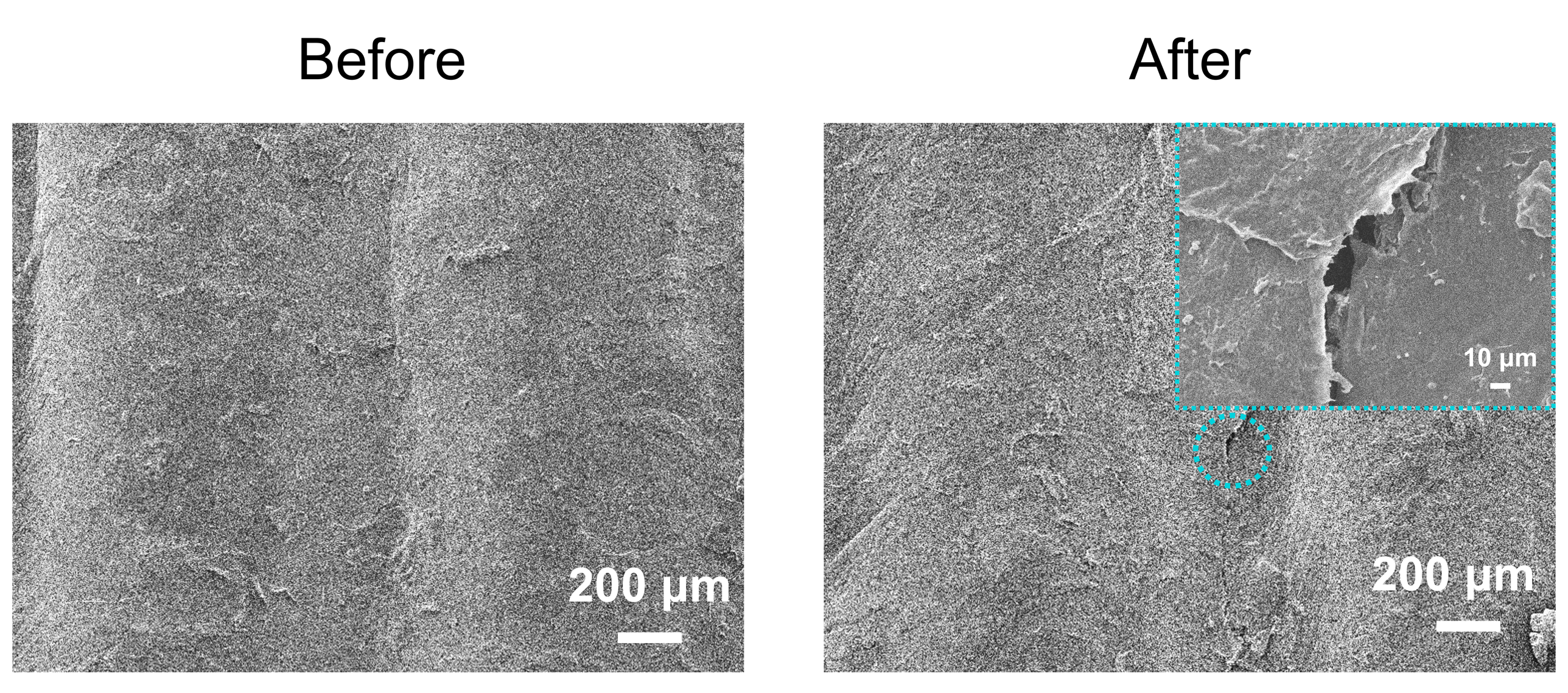

Supplement: Supplementary 1 — Figs. S1 to S24 Tables S1 to S5 Movies S1 to S4 [file research.1358.f1.zip › Fig. S9.tif]
